# Olanzapine Treatment of Adolescent Rats Causes Enduring Specific Memory Impairments and Alters Cortical Development and Function

**DOI:** 10.1371/journal.pone.0057308

**Published:** 2013-02-20

**Authors:** Jean A. Milstein, Ahmed Elnabawi, Monika Vinish, Thomas Swanson, Jennifer K. Enos, Aileen M. Bailey, Bryan Kolb, Douglas O. Frost

**Affiliations:** 1 Dept. of Pharmacology, University of Maryland School of Medicine, Baltimore, Maryland, United States of America; 2 Dept. of Epidemiology and Public Health, University of Maryland School of Medicine, Baltimore, Maryland, United States of America; 3 Dept. of Psychology, St. Mary's College of Maryland, St. Mary's, Maryland, United States of America; 4 University of Lethbridge, Canadian Center for Behavioral Neuroscience, Lethbridge, Alberta, Canada; 5 Dept. of Psychiatry, University of Maryland School of Medicine, Baltimore, Maryland, United States of America; 6 Program in Neuroscience, University of Maryland School of Medicine, Baltimore, Maryland, United States of America; University of Queensland, Australia

## Abstract

Antipsychotic drugs are increasingly used in children and adolescents to treat a variety of psychiatric disorders. However, little is known about the long-term effects of early life antipsychotic drug treatment. Most antipsychotic drugs are potent antagonists or partial agonists of dopamine D2 receptors; atypical antipsychotic drugs also antagonize type 2A serotonin receptors. Dopamine and serotonin regulate many neurodevelopmental processes. Thus, early life antipsychotic drug treatment can, potentially, perturb these processes, causing long-term behavioral- and neurobiological impairments. Here, we treated adolescent, male rats with olanzapine on post-natal days 28–49. As adults, they exhibited impaired working memory, but normal spatial memory, as compared to vehicle-treated control rats. They also showed a deficit in extinction of fear conditioning. Measures of motor activity and skill, habituation to an open field, and affect were normal. In the orbital- and medial prefrontal cortices, parietal cortex, nucleus accumbens core and dentate gyrus, adolescent olanzapine treatment altered the developmental dynamics and mature values of dendritic spine density in a region-specific manner. Measures of motor activity and skill, habituation to an open field, and affect were normal. In the orbital- and medial prefrontal cortices, D1 binding was reduced and binding of GABA_A_ receptors with open Cl^−^ channels was increased. In medial prefrontal cortex, D2 binding was also increased. The persistence of these changes underscores the importance of improved understanding of the enduring sequelae of pediatric APD treatment as a basis for weighing the benefits and risks of adolescent antipsychotic drug therapy, especially prophylactic treatment in high risk, asymptomatic patients. The long-term changes in neurotransmitter receptor binding and neural circuitry induced by adolescent APD treatment may also cause enduring changes in behavioral- and neurobiological responses to other therapeutic- or illicit psychotropic drugs.

## Introduction

There is increasing awareness among the clinical and research communities of the potential for long-term sequelae arising from psychotropic medication of pediatric patients. Antidepressants have been most widely studied in this regard [1], [2], whereas antipsychotic drugs (APDs) have been investigated much less. Although APDs vary in the spectra of their pharmacologic actions, nearly all APDs potently inhibit dopamine type 2 (D2) family receptor function; atypical APDs also have a broad spectrum of serotonergic (5HTergic) and other activities [3], [4]. Some of the 5HTergic activities contribute to the therapeutic efficacy of the atypical APDs clozapine and olanzapine (OLA) [5]. Atypical APDs, including OLA, are commonly prescribed to treat multiple psychiatric disorders in pediatric patients [6], [7], [8], [9], [10], [11]. Patients with psychotic illness often require long-term maintenance therapy, but most pediatric APD treatment is for other, off-label indications [12], [13], for which consensus guidelines [14], [15], [16] recommend trial off-medication after stability is achieved. Although short-term tolerance of APDs is reasonably good in adult and pediatric patients [17], the long-term sequelae that follow cessation of APD treatment in pediatric patients are unknown [9]. In the mature brain, dopaminergic (DAergic) signaling is one of the mechanisms of cognition, learning and memory [18], [19]. Monoamines, including DA and 5HT, also regulate a variety of neurodevelopmental processes [20], [21], [22]. Thus, even time-limited APD therapy can, potentially, alter the subsequent developmental trajectory of the brain, resulting in long-term abnormalities of brain function and behavior. In humans, the cerebral substrates upon which APDs act undergo dramatic ontogenetic changes into the third decade [23], [24]. This raises the possibility that the behavioral and neurobiological sequelae of early life APD treatment, could vary with age of treatment, and underscores the necessity of independently evaluating the enduring sequelae of APD treatment in humans and in animal models at different ontogenetic stages.

Previous studies of the long-term behavioral effects of adolescent APD treatment in animal models have not revealed any long-term cognitive deficits [25], [26], [27], although a deficit of working memory has been demonstrated in adolescence, shortly after the cessation of APD administration [28]. Similarly, the impact of adolescent APD treatment on receptor binding, an important determinant of synaptic function, has been studied only at the end of treatment [29], [30], [31]. To our knowledge, there have been no studies of the impact of adolescent APD treatment on the development and mature configuration of “hard wired” neural circuitry. The present experiments are designed to elucidate the *long-term* effects of adolescent OLA treatment. We focused on memory functions mediated by the prefrontal cortex and hippocampus, and neurobiological changes in those regions, because the hippocampus and prefrontal cortex receive a robust DAergic innervation and develop until late adolescence. We also studied additional behaviors that are directly or indirectly modulated by DAergic function. We found that adult rats treated with OLA during a limited period of adolescence exhibit persistent learning deficits, accompanied, in behaviorally relevant brain regions, by abnormalities of DA- and GABA_A_ receptor binding and alterations of dendritic form, a key determinant of the functional architecture of neural circuitry.

## Materials and Methods

### Ethics Statement

These experiments were conducted in accordance with the recommendations in the Guide for the Care and Use of Laboratory Animals of the National Institutes of Health. Our protocols were approved by the Institutional Animal Care and Use Committees of the University of Maryland, Baltimore (Protocol #0411003), St. Mary's College of Maryland (Protocol #R010907) and the University of Lethbridge (Protocol #0712).

### Subjects

We studied male, colony-bred Long-Evans rats (breeding stock obtained from Charles River, Wilmington, MA). Litters were culled to 10–12 pups on postnatal day 7 (P7; first 24 h after birth  =  P0). Rats were maintained under standard laboratory conditions on a light cycle of 14 h light/10 h dark, except as noted below. Water and food were available *ad libitum*. On P21, subjects were weaned and pair- or triple-housed with same-sex littermates.

### Drug Treatments

In other studies [32], we administered OLA in the drinking water at a target dose of 7.5 mg/kg/d, starting on P28. On P49, plasma OLA concentrations at mid-dark- and mid-light- phases of the diurnal cycle, which correspond, respectively, to periods of high- and low activity (and drinking), were 93.0±28.5 and 16.2±12.2 ng/ml (mean±SD). These values approximate a broad spectrum of steady state plasma OLA levels (135.7±91.0 ng/ml; mean±SD) that, *in rats*, produces mean D2 receptor occupancies in the human therapeutic range [33]. In rats, lower- or higher OLA doses produce receptor occupancies outside this range ([33], [34]; higher doses could also cause catalepsy).

For the animals studied in the present experiments, on P28–49 the drinking water was replaced with an aqueous solution of OLA in 1 mM acetic acid or vehicle (VEH), alone. The OLA solution was mixed fresh daily at a concentration calculated to deliver a target dose of 7.5 mg/kg/d, based on the weights and water consumption of the rats over the previous 24 h. This regimen achieved ∼96% of the target dose. On P50, all rats were switched to normal drinking water. This procedure was designed to reproduce as closely as possible the clinical administration of atypical APDs. We [32] and others [28] have shown that, in contrast to the effects of OLA in humans and in adult rats, *adolescent* OLA treatment in rats does not significantly increase weight or alter plasma glucose levels [28] during or after treatment; triglyceride levels are unchanged during adolescent OLA treatment and fall following termination of treatment [28]. Thus, the behavioral and neurobiological effects reported here for rats treated with OLA as adolescents occur in the absence of the metabolic changes induced by atypical APDs in humans and adult rats.

### Distribution of assays

**Figure 1:**
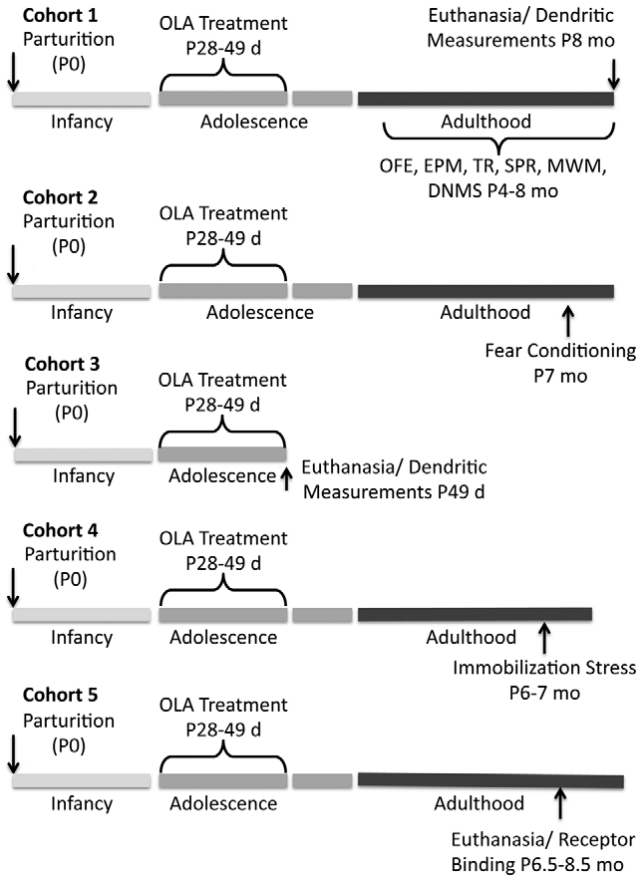
Experimental cohorts. Time lines illustrate, for each cohort, the timing of OLA treatment and the experimental measures obtained. Abbreviations: OFE - open field exploration; EPM - elevated plus maze; TR - tray reaching; SPR single pellet reaching; MWM - Morris water maze; DNMS - delayed non-match to sample

It is not feasible to obtain all our experimental measures in the each subject. Thus, we studied 5 separate cohorts of rats (Fig. 1):

1) 11 OLA-treated and 9 vehicle-treated [VEH] control rats, studied over ages 4–8 months, were tested in the following sequence of behavioral tasks: i) open field exploration, ii) elevated plus maze, iii) skilled reaching, iv) Morris water maze and v) delayed non-match to sample. At the completion of testing (age 8 mo) all 11 OLA-treated rats and 7 of the 9 VEH-treated rats were euthanized and their brains were processed for Golgi histology to assess dendritic architecture at maturity, when the behavioral testing was done.

2) 11 OLA-treated and 8 vehicle-treated [VEH] control rats were tested for fear conditioning/extinction at age 7 months.

3) Six OLA-treated and 6 VEH-treated control rats were euthanized on P49 and their brains were processed for Golgi histology to study the effects of OLA on dendritic form, while it was present in the brain.

4) 13 OLA-treated and 11 VEH-treated rats were used to investigate the corticosterone response to mild immobilization stress at ages 6–7 months.

5) Receptor binding measures were obtained from a fifth cohort (9 OLA-treated and 9 vehicle-treated [VEH] controls) at age 6–8.5 months. D1 and D2 receptor binding in the prefrontal cortex reaches mature levels by P100 [35], [36], [37] and *in vivo* PET imaging shows that D1 and D2 receptor binding levels in adult rats do not begin their age-associated decline until some time between ages 12 and 24 months [38]. Thus, our data are not confounded by normal developmental- or aging-associated changes in DA receptor binding.

In each cohort, each treatment group was drawn from members of ≥3 litters and members of each litter were included in each treatment group, to avoid uncontrolled litter and age effects.

All protocols were in accordance with the National Research Council Guide for the Care and Use of Laboratory Animals and approved by our institutional animal care and use committees.

### Behavioral Testing

Behavioral tests were conducted during the light phase of the diurnal cycle (12 h light, 12 h dark).

#### Working memory - delayed non-match to sample (DNMS) test

Rats were food restricted to 90% of *ad libitum* body weight prior to testing and maintained at this weight until the end of the test when *ad libitum* food was restored. First, they were habituated to the T-maze apparatus. Rats were then trained to run to the ends of the maze arms to obtain a food reward (fruit loops), which was provided regardless of the arm they entered. Testing began once all the rats readily entered the arms to retrieve a reward. The rats were required to learn a delayed non-match to sample (“rewarded alternation”) task [39]. Each trial consisted of two components. In the first, one arm was blocked, forcing the subject into the open arm. In the second, the block was removed and the rat was reintroduced to the maze (10 s delay). To retrieve a reward, the subject had to choose the arm opposite the one in which the reward had been obtained in component one. Subsequent trials began about 30 s after the end of the second component. Each subject received 10 trials/day, for 10 consecutive days. The significance of inter-group performance measures was analyzed using independent, 2-tailed t-tests.

#### Spatial memory - Morris water maze (MWM) test

Spatial memory was assessed over 10 consecutive days using the “fixed platform” version of the MWM (days 1–5), followed by testing on the “moving platform” version (days 6–10). The apparatus was a round, polyethylene pool (2 m diameter). The escape platform was hidden approximately 2 cm below the surface of the opaque water (25°C). In the fixed platform stage the rats received 4 trials/day (1 trial started in each quadrant, daily, in random order) in which the hidden platform had a fixed location, as previously described [40]. For the moving platform assay, platform location was changed every day. The first trial of each day was considered an information trial, in which the rat learned the location of the platform. The next 3 trial latencies were averaged for each rat to assess working memory. Distance traveled before finding the platform, swim speed, and escape latency were recorded for each trial using a video tracking system. Latency and path length were analyzed by repeated measures ANOVA and Bonferroni-corrected, paired comparisons; swim speed was analyzed by an independent t-test.

#### Acquisition/extinction of fear conditioning

Pavlovian fear conditioning and extinction were measured over two days. Rats were placed in a standard fear conditioning chamber (30.5 cm L×24.1 cm W×21.0 cm H; Med Associates, St. Albans, VT) and trained to associate a tone (conditional stimulus [CS]; 4 kHz, 80db SPL, 30 s duration) with a 0.7 mA scrambled foot shock (unconditional stimulus; US). On Day 1, rats were placed in the chamber and allowed to habituate for 120 s. They were then given two presentations of the CS alone, to measure pre-conditioning responses to the tone, followed by seven CS-US pairings (120 s inter-trial interval [ITI]). All CS presentations were 30 s; the 1 s US co-terminated with the CS. Twenty-four hours after the original conditioning, all rats were returned to the same chamber and were given 15 presentations of the CS alone (120 s ITI), to measure extinction of conditioned fear. The total duration of freezing behavior during the presentation of each CS was measured using a computer-assisted video analysis system (Med Associates), expressed as a percentage of the CS duration and analyzed by repeated measures ANOVA and Bonferroni-corrected, paired comparisons.

### Dendritic Architecture

Quantitative measures of dendritic form were obtained from the rats of cohort (a), euthanized as adults (at age ∼8 months) by overdose with Uthansol (100 mg/kg, IP), and from cohort (b), euthanized on P49 at the completion of OLA or VEH treatment. Their brains were removed from the skull, processed by the Golgi-Cox technique, sectioned on a vibratome at 200 microns and mounted onto slides, as previously described [41]. Using a microscope equipped with a 25X objective and a camera lucida, or a 60X objective on a computer-assisted microscope, we made flattened reconstructions of the dendritic arbors of layer 3 pyramidal cells of the orbital and medial prefrontal cortices (OPC and MPC (AID and Cg3; [42]), respectively) and parietal (primary somatosensory) cortex (PAR1; [42]), medium spiny neurons (MSNs) of the NAc core, and granule cells (GCs) of the dentate gyrus (DG) of the *dorsal* hippocampus. From these, we determined total dendritic length and the number of dendritic segments (a measure of branching complexity). Using a 100X objective, we also determined dendritic spine density (a measure of the number of excitatory synapses converging on individual neurons). At least for stimulants, spine density on peripheral dendritic segments is more responsive to drug treatment than spine density on proximal segments [43]. Thus, we sampled spine density over third order segments of pyramidal cell basal dendrites and MSNs and on the most distal segments of pyramidal cell apical dendrites and GCs. All measures of dendritic form were determined for ≥6 neurons/region of interest (ROI) in each brain. Each ROI was analyzed separately. For each subject, the average of each measure across the 6 reconstructed cells was used for statistical analysis of the effects of adolescent treatment, age and their interaction (i.e., n is the number of rats; 2-way ANOVA). The Bonferroni correction was applied to p-values obtained in independent, pair-wise t-tests. We also analyzed the number of dendritic segments of each order by repeated measures ANOVA. In DG, for technical reasons, on P49, only total dendritic length was measured.

### Receptor Binding

Nine OLA-treated and 9 control rats from cohort (e) were euthanized by asphyxiation with CO_2_. Their brains were removed and punches of tissue were obtained from each ROI (selected on the basis of its relevance to our behavioral tests and its robust DAergic innervation). For each ROI, punches from the two sides of the brain in the same individual were pooled, snap frozen in an isopentane/dry ice mix and stored at −80°C until processing. In order to obtain large enough samples for our assays, tissue from 3 rats was pooled for each of the 3 independent samples studied for each treatment group (i.e., for statistical purposes n = 3/group).

Cell membranes were prepared at 4°C [44]. Briefly, tissue punches were homogenized in Tris-Krebs Ringer (pH 7.4, 118 mM NaCl, 4.8 KCl, 2.5 mM CaCl_2_, 1.2 mM MgSO_4_, 1 mM EDTA) and the homogenate was centrifuged (40,000×g, 20 min). The resultant pellet was resuspended in 50 mM Tris-HCl, pH 7.4, then centrifuged at (40,000×g, 20 min). The final pellet was resuspended in 50 mM Tris-HCl buffer, pH 7.4, and kept cold until protein content was determined using BCA reagents (bovine serum albumin standard).

Binding of [^3^H]SCH-23390 (70.30 Ci/mmol; 3 nM) and [^3^H]YM-09151-2 (85.50 Ci/mmol; 2.5 nM) to D1-family and D2-family receptors, respectively, was determined by filtration assay [44]. For D1 binding, membrane homogenates were incubated with [^3^H]SCH-23390 in 50 mM Tris-HCl buffer, pH 7.4, at 23°C (1 h). Non-specific binding of [^3^H]SCH-23390 was measured in the presence of 100 µM flupenthixol. All tubes contained 40 nM ketanserin to preclude binding to 5-HT2 receptors. For D2 binding, homogenates were incubated with [^3^H]YM-09151-2 in 50 mM Tris-HCl buffer, pH 7.4, containing 120 mM NaCl at 23°C (1 h). Non-specific binding of [^3^H]YM-09151-2 was measured in the presence of 100 µM spiperone. All tubes contain 10 nM ketanserin to preclude binding to 5-HT2 receptors. For GABA_A_ binding, homogenates were incubated with [^3^H] ethynylbicycloorthobenzoate (EBOB; 30 Ci/mmol; 6.5 nM) in 50 mM Tris-HCl buffer, pH 7.4, containing 300 mM NaCl at 23°C (1 h). Non-specific binding of [^3^H]EBOB was measured in the presence of 100 µM picrotoxin. Bound- and free ligands were separated by rapid filtration over Whatman GF/B glass-fiber filters (presoaked in 0.05% polyethylenimine for ≥20 min), then washed with ice-cold 0.9% NaCl (3×4 ml). Radioactivity was counted by liquid scintillation spectroscopy. All samples were run in triplicate; the mean of the 3 measures was used for statistical analyses (2-tailed t-test).

## Results

### Learning and Memory

#### Working memory (DNMS)

**Figure 2:**
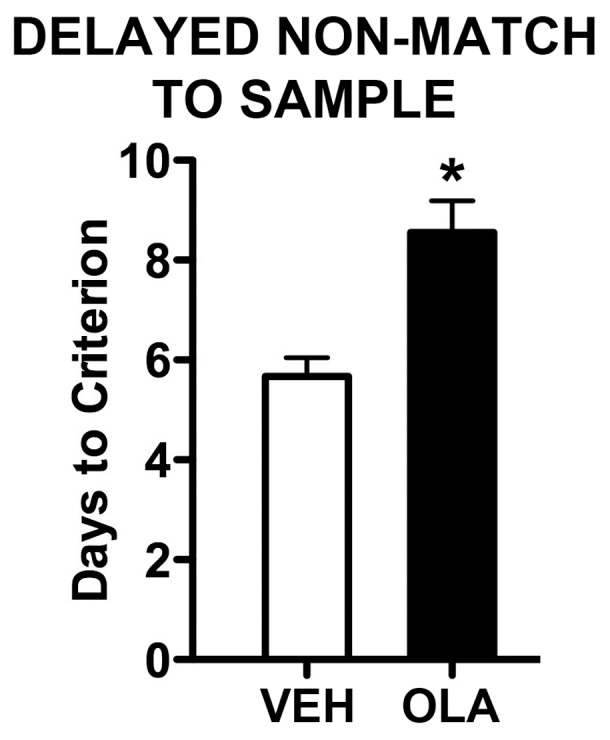
Delayed non-match to sample performance. Number of days required to reach criterion performance (≥80% correct on 3 consecutive days). Error bars are standard error of the mean. * indicates p = 0.007.

Seven (64%) of the OLA-treated rats and 9 (100%) of the VEH-treated rats attained criterion performance (≤80% correct on 3 consecutive days) by the last (tenth) day of testing. For statistical analysis, of the rats that did not reach criterion, two that performed at ≤80% correct on day 10 were designated as attaining criterion on day 12 and two that still performed at <80% correct on day 10 were designated as attaining criterion on day 13 – the earliest days on which they might have reached criterion had they been tested longer. Despite this conservative scoring procedure designed to favor the null hypothesis, OLA-treated rats still took significantly longer than controls to learn the DNMS task (Fig. 2; t[18] = 3.67, p = 0.007).

#### Spatial memory (MWM)

**Figure 3:**
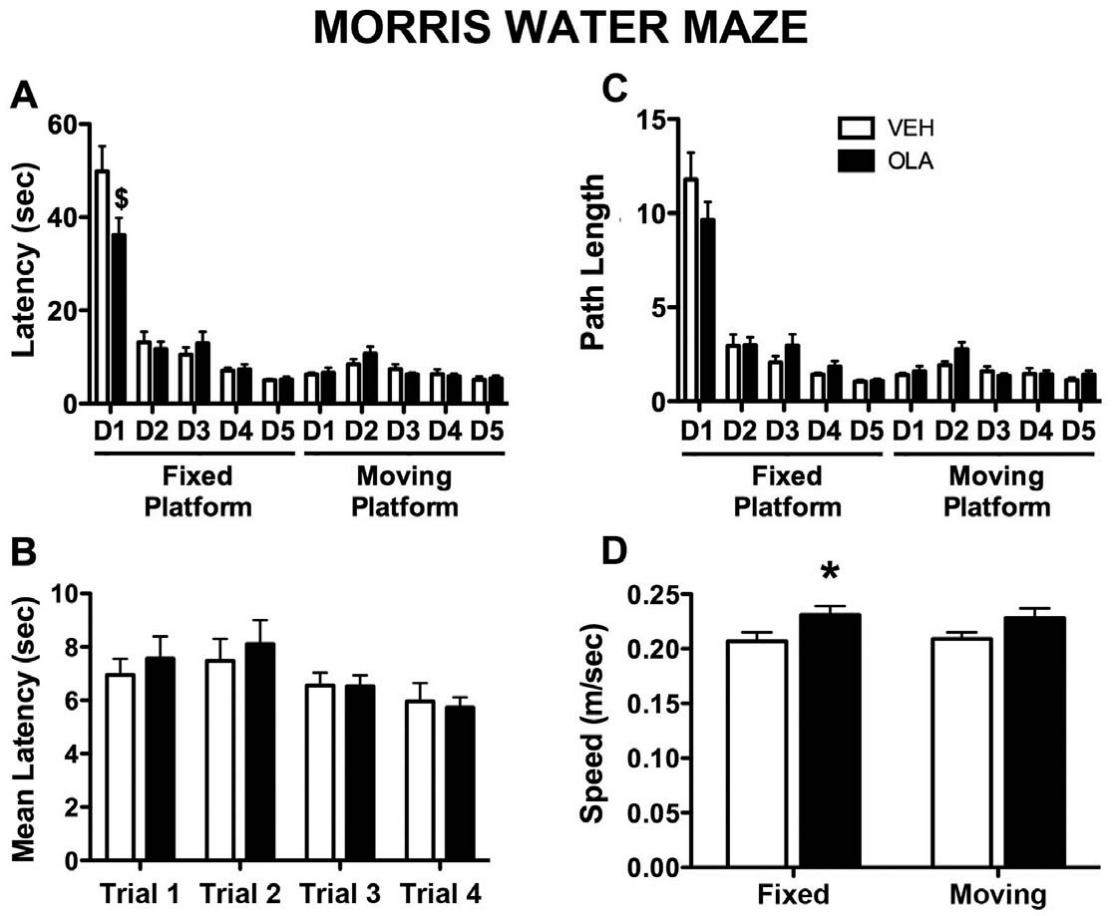
Morris water maze performance. A. Latency to find the hidden platform on each day of testing; the measure used is the average latency in the 4 trials on that day. D1–D5 indicate days 1–5 of either the fixed- or moving platform versions of the MWM test. OLA treatment significantly altered MWM performance on the first day of testing only ($). B) Latency to find the hidden platform as a function of trial number, averaged across all 5 days of “moving platform” testing. There was no significant effect of OLA treatment on any trial. C. Path length during the search for the hidden platform on each day of testing; the measure used is the average path length in the 4 trials on that day. D. Mean swim speed averaged across trials and days of testing for each rat, with data from the fixed- and moving platform versions of the MWM analyzed separately. Error bars are standard error of the mean. * indicates p = 0.05.

In the fixed- platform version of the MWM, there was a significant effect of test session on latency to find the hidden platform (Fig. 3A; F(4,72) = 89.554, p<0.001) and on path length (Fig. 3C; F(4,72) = 94.309, p<0.001). When the latency data from the moving platform test are pooled over days and grouped by trial, there are no significant effects of trial, OLA treatment or their interaction (Fig. 3B, p>0.05 for all measures). OLA treatment increased swim speed to ∼1.1 times that in VEH-treated rats (Fig. 3D; fixed platform: F[1,18] = 4.376, p = 0.05; moving platform: F[1,18] = 2.705, p = 0.117; fixed and moving platform data pooled: F[1,18] = 5.303, p = 0.033) but had no significant effect on latency or path length (Fig. 3A,C; p>0.05 for both measures, in either the fixed- or moving platform versions of the MWM). There were no significant interactions of treatment and test session except for latency in the fixed platform version of the test (Fig. 3A,C; F[4,18] = 3.893, p = 0.006). This effect is driven by the data from the first test session; it is not significant if data from that session are eliminated and there is no significant effect of treatment in pair-wise t-tests on the data from individual sessions 2–10. Thus, despite their enduring deficit in working memory in the DNMS task, adult rats treated with OLA as adolescents do not have a deficit of spatial memory in the MWM.

#### Acquisition/extinction of fear conditioning

**Figure 4:**
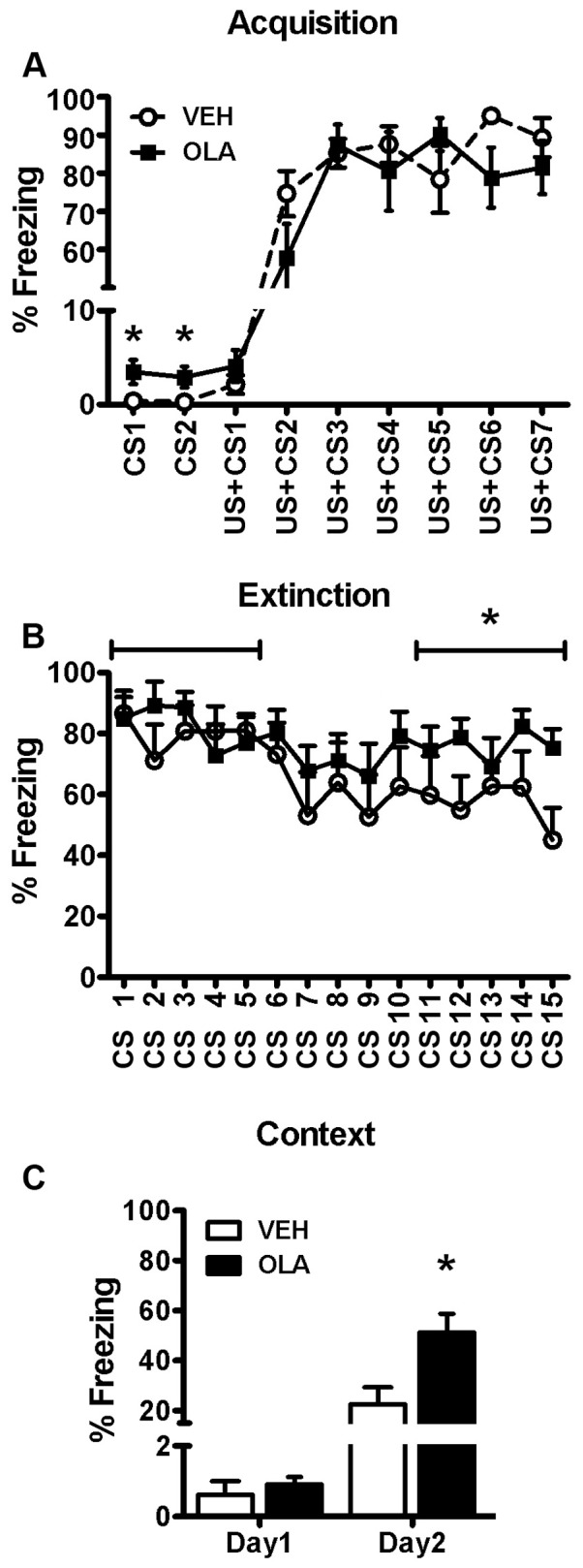
Fear conditioning and extinction to cue (A and B) and context (C). A. Acquisition (Day 1). Amount of freezing in response to two presentations of the conditional stimulus alone (CS), followed by 7 paired presentations of the CS and the unconditional stimulus (US+CS). B. Extinction (Day 2). Amount of freezing in response to 15 presentations of the conditional stimulus alone. Horizontal lines define the two blocks of trial for which mean freezing was calculated for each group and then studied in a supplemental analysis (see text). * indicates that for the block consisting of the last 5 trials, there was a significant treatment effect, although there was no significant treatment effect for the first block. C. Amount of freezing during the first 120 s after rats were placed in the fear conditioning chamber, prior to any stimulus presentations. Units on the vertical axis are the percentage of the CS presentation time (30 s; A and B) or the percentage of the first 120 sec after rats were placed in the fear conditioning chamber, prior to any stimulus presentations (C), during which freezing occurred.

On Day 1, the durations of freezing responses to the two unpaired CS presentations were analyzed by 2-way repeated measures ANOVA (treatment X trial). There was no overall effect of trial (F[1,17] = 0.17, p>0.05) and no interaction between treatment and trial (F[1,17] = 0.04, p>0.05). However, the OLA-treated rats spent significantly more time freezing during the CS (3.2%) than VEH-treated animals (0.3%), (F[1,17] = 6.6, p = 0.02; Fig. 4A). Acquisition of conditioned freezing responses was analyzed for the 7 CS-US trials using a separate, 2-way repeated measures ANOVA (treatment X trial). There was an overall increase in freezing with repeated trials (F[6,102] = 50.11, p<0.001) but no overall effect of treatment ([F1,17] = 0.93, p>0.05) and no treatment X trial interaction (F[6,102] = 1.44, p>0.05).

Extinction of the CS-US association was investigated on Day 2 by analyzing the percentage of time spent freezing during each of the 15 presentations of the CS alone (Fig. 4B). There was an overall decrease in freezing across trials (F[14,238] = 2.376, p = 0.004) but no overall effect of treatment (F[1,17] = 2.12, p = 0.16) and no treatment X trial interaction (F[14,238] = 0.87, p>0.05). However, planned comparisons showed that, for VEH-treated animals, there was a significant decrease in freezing between the first- (86.4±5.5%) and last (44.9±10.6%) extinction trials (t[7] = 6.82, p<0.001) whereas for OLA-treated rats, there was no significant change (84.9±8.9% and 75.1±6.3% for the first- and last trials, respectively; t[10] = 1.39, p = 0.195).

Perusal of Fig. 4B shows that, for approximately the first half of the 15-trial sequence on Day 2, neither treatment group showed clear signs of extinction. This might have caused a type 2 (false negative) error in assessing the main effect of treatment, because the ANOVA was conducted over the entire sequence. To address this issue, we performed a supplementary ANOVA of freezing averaged over two blocks consisting of the first 5 and last 5 extinction trials. We found a significant overall effect of block (F[1,17] = 10.34, p = 0.005), a trend-level treatment X block interaction, (F[1,17] = 3.23, p = 0.09) but no overall treatment effect, (F[1,17] = 2.38, p = 0.14). Post-hoc analysis (α = 0.025) indicated that there was no significant difference in the amount of freezing by OLA- and VEH-treated rats during the first block, (t[17] = 0.27, p = 0.79), although OLA-treated animals froze significantly more than VEH-treated rats during the last block (t[17] = 2.6, p = 0.02).

On Days 1 and 2, we also measured contextual freezing during the first 120 s that the rats were in the test chamber, prior to the first CS presentation. From Day 1 to Day 2, there was a significant overall increase in contextual freezing (Fig. 4C; F[1,17] = 52.24, p<0.001). Overall, OLA-treated rats spent significantly more time in contextual freezing than VEH-treated, control rats (F[1,17] = 6.85; p = 0.018) and there was a significant treatment X day interaction (F[1,17] = 6.51, p = 0.021). On Day 1, the OLA- and VEH-treated rats did not differ significantly in contextual freezing, (t[17] = 1.13, p = 0.28). However, on Day 2, OLA-treated rats spent significantly more time in contextual freezing than VEH-treated rats (t[17] = 2.59, p = 0.019).

### Other Behavioral Domains

#### Activity and motor performance

Adolescent OLA treatment has no significant effects on multiple measures of open field exploratory activity assessed in adulthood (Fig. S1). This suggests normal responsiveness to a novel environment and no gross motor deficit. The absence of motor effects is further supported by our data showing that the same rats also are normal in their performance on two skilled reaching tasks (Fig. S2).

#### Affective measures

Adolescent OLA treatment had no significant effect on time spent in the open arms of an elevated plus maze, a behavioral measure of anxiety in rodents (Fig. S3) or on multiple measures of corticosterone (CORT) release in response to a mild transient stress, disruption of which is a phenotype of affective disorders (Fig. S4).

### Dendritic Architecture

**Figure 5:**
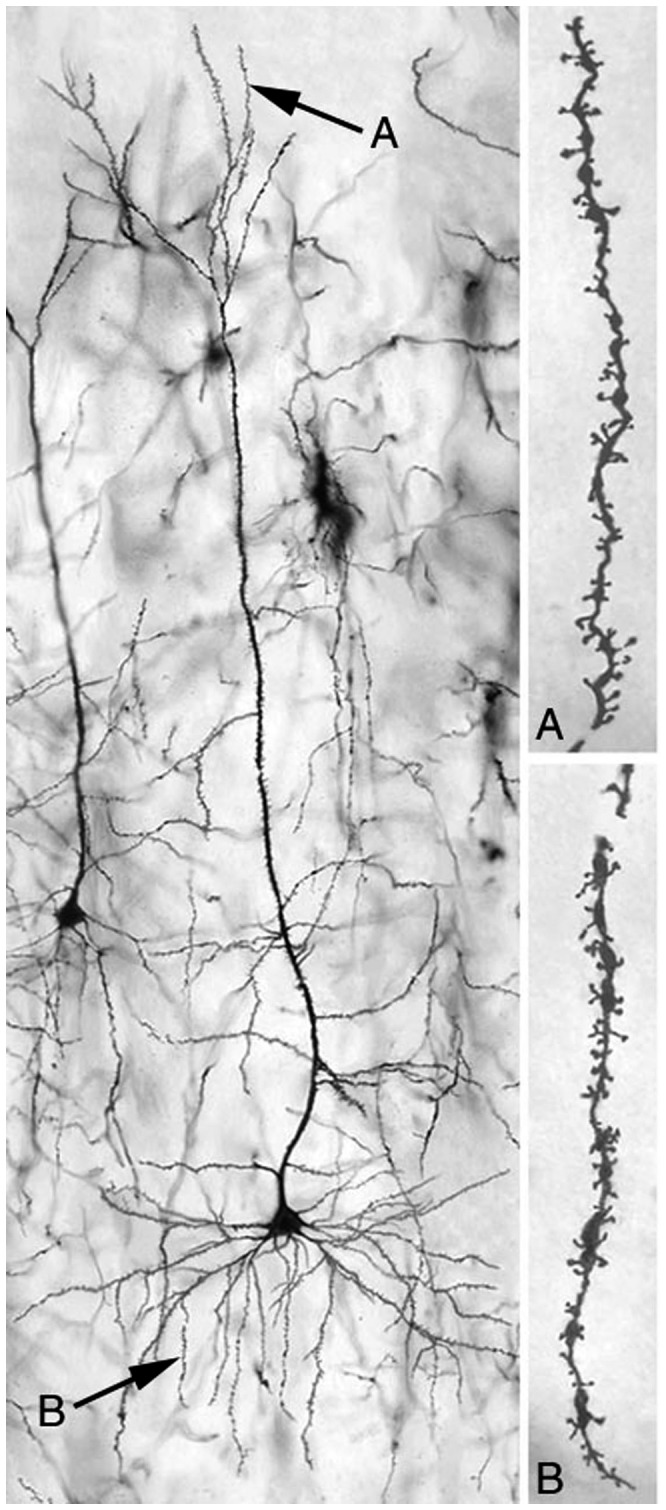
Photomicrograph of a typical layer 3 pyramidal cell in MPC. “A” and “B” indicate, respectively, distal apical dendrites and third order basal dendrites, the regions from which dendritic spine density measures were obtained.

We quantitatively analyzed the dendritic architecture of layer 3 pyramidal cells in OPC, MPC and PAR1, dentate granule cells, and MSNs of the NAc core, as described above. The spectrum of effects of OLA treatment, age and their interaction were unique for each ROI (Table 1). A typical Golgi-filled pyramidal cell is shown in Fig. 5.

**Table 1:** Main effects of age, treatment and age X treatment interaction on dendritic architecture.

DENDRITIC SPINE DENSITY	MPC Apical	MPC Basal	OPC Basal	PAR1 Apical	PAR1 Basal	NAc Core (Radially Symmetric)	DG (Unipolar, Adult Only)
Age Effect	F(1,26) = 72.08; p<0.001	F(1,26) = 94.547; p<0.001	F(1,26) = 1.235; p = 0.277	F(1,26) = 97.5; p<0.001	F(1,26) = 161.7; p<0.001	F(1,26) = 29.451 p<0.001	NA
Treatment Effect	F(1,26) = 99.16; p<0.001	F(1,26) = 42.219; p<0.001	F(1,26) = 9.58; p = 0.005	F(1,26) = 3.27; p = 0.082	F(1,26) = 2.81; p = 0.106	F(1,26) = 3.595; p = 0.069	F(1,17) = 4.421; p<0.001
Age X Treatment Interaction	F(1,26) = 108.28; p<0.001	F(1,26) = 120.190; p<0.001	F(1,26) = 40.6; p<0.001	F(1,26) = 3.27; p = 0.087	F(1,26) = 2.70; p = 0.113	F(1,26) = 3.267; p = 0.082	NA
TOTAL DENDRITIC LENGTH	MPC Apical	MPC Basal	OPC Basal	PAR1 Apical	PAR1 Basal	NAc Core (Radially Symmetric)	DG (Unipolar)
Age Effect	F(1,26) = 48.47; p<0.001	F(1,26) = 499.04; p<0.001	F(1,26) = 1.441; p = 0.241	F(1,26) = 0.245; p = 0.625	F(1,26) = 17.89; p<0.001	F(1,26) = 16.7; p<0.001	F(1,26) = 94.778; p<0.001
Treatment Effect	F(1,26) = 1.35; p = 0.25	F(1,26) = 1.71; p = 0.202	F(1,26) = 2.35; p = 0.137	F(1,26) = 0.337; p = 0.567	F(1,26) = 0.049; p = 0.826	F(1,26) = 0.401; p = 0.532	F(1,26) = 0.372; p = 0.547
Age X Treatment Interaction	F(1,26) = 0.001; p = 0.97	F(1,26) = 7.36; p = 0.012	F(1,26) = 6.66; p = 0.016	F(1,26) = 0.002; p = 0.960	F(1,26) = 0.578; p = 0.454	F(1,26) = 0.856; p = 0.363	F(1,26) = 3.428; p = 0.075

TOTAL NUMBER OF DENDRITIC SEGMENTS	MPC Apical	MPC Basal	OPC Basal	PAR1 Apical	PAR1 Basal	NAc Core (Radially Symmetric)	DG (Unipolar, Adult Only)
Age Effect	F(1,26) = 71.998; p<0.001	F(1,26) = 124.3; p<0.001	F(1,26) = 28.19; p<0.001	F(1,26) = 149.55; p<0.001	F(1,26) = 32.693; p<0.001	F(1,26) = 49.5; p<0.001	NA
Treatment Effect	F(1,26) = 2.244; p = 0.146	F(1,26) = 0.31; p = 0.583	F(1,26) = 1.82; p = 0.189	F(1,26) = 3.089; p = 0.091	F(1,26) = 6.113; p = 0.020	F(1,26) = 0.708; p = 0.408	F(1,17) = 0.029; p = 0.866
Age X Treatment Interaction	F(1,26) = 0.216; p = 0.646	F(1,26) = 0.581; p = 0.453	F(1,26) = .239; p = 0.629	F(1,26) = 6.039; p = 0.021	F(1,26) = 4.906; p = 0.036	F(1,26) = 0.001; p = 0.97	NA

Cells in bold contain statistically significant effects. In the NAc core and dentate gyrus, neurons are radially symmetric and unipolar, respectively, so there is no distinction of apical and basal dendrites. ‘ND’ indicates statistics based on measures that were not determined in the DG for technical reasons.

#### Dendritic spine density

**Figure 6:**
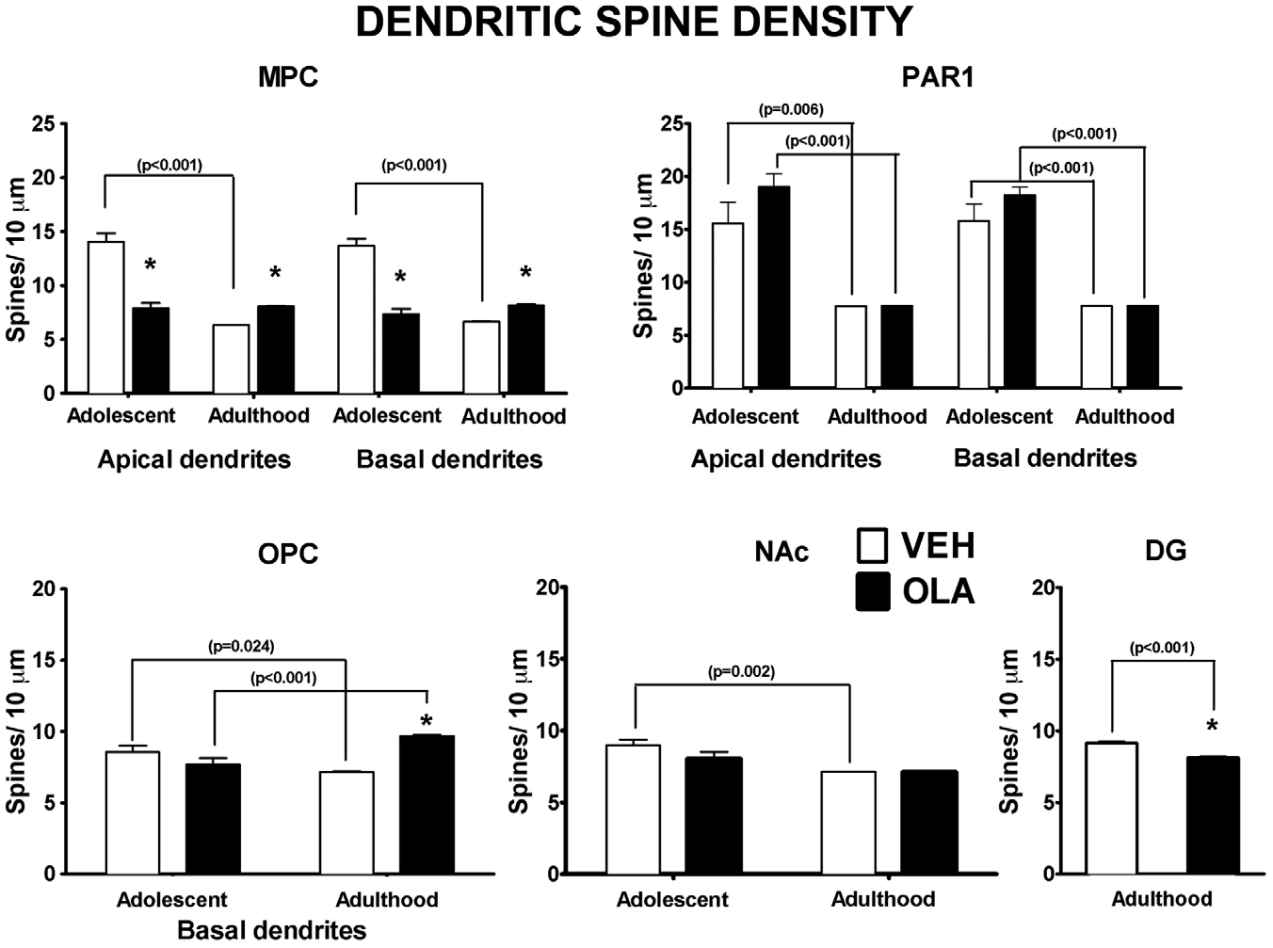
Dendritic spine density. * indicates statistically significant difference (p = 0.024 to <0.001) between VEH- and OLA-treated rats at the same age. p-values above brackets indicate the significance of age-related changes in similarly treated rats. All p-values are based on paired t-tests corrected for multiple comparisons. Error bars are standard error of the mean.

In VEH-treated control rats, there is a reduction in spine density between mid-adolescence (P49) and adulthood (measured at ∼8 months old; Fig. 6, Table 1): by ∼50% in PAR1 and MPC, ∼16% in OPC and ∼21% in the NAc core. In PAR1 and MPC, apical and basal dendrites exhibit similar age effects. (For technical reasons, we were unable to obtain dendritic spine density data in DG of adolescent rats. We studied only basal dendrites in OPC, as the apical dendrites were usually truncated due to the plane of sectioning).

OLA treatment on P28–49 alters the developmental dynamics of dendritic spine density between the cessation of treatment and adulthood in MPC and OPC (but not in PAR1), and in the NAc core (Fig. 6, Table 1). The effects are regionally specific. In MPC of OLA-treated rats, spine density is the same at P49 and at maturity, at a level that is intermediate between the normal density on P49 and the lower density in normal adults. Thus, spine density is ∼57% of normal on P49 and 125% of normal at maturity. In the OPC of OLA-treated rats, dendritic spine density increases between P49 and maturity, rather than decreasing as in VEH-treated controls. Thus, although spine density in OLA-treated rats does not differ significantly from normal on P49, it is 135% of normal in adulthood. In PAR1 and NAc of OLA-treated rats, spine density falls between P49 and maturity in both regions, as it does in VEH-treated rats, although, in the NAc, this decrease is blunted to a trend level (t[15] = 2.71; p = 0.063). There are no significant effects of OLA treatment and no significant interactions between age and treatment in PAR1 or NAc. In DG of OLA-treated rats, spine density is ∼89% of normal in adults.

#### Total dendritic length

**Figure 7:**
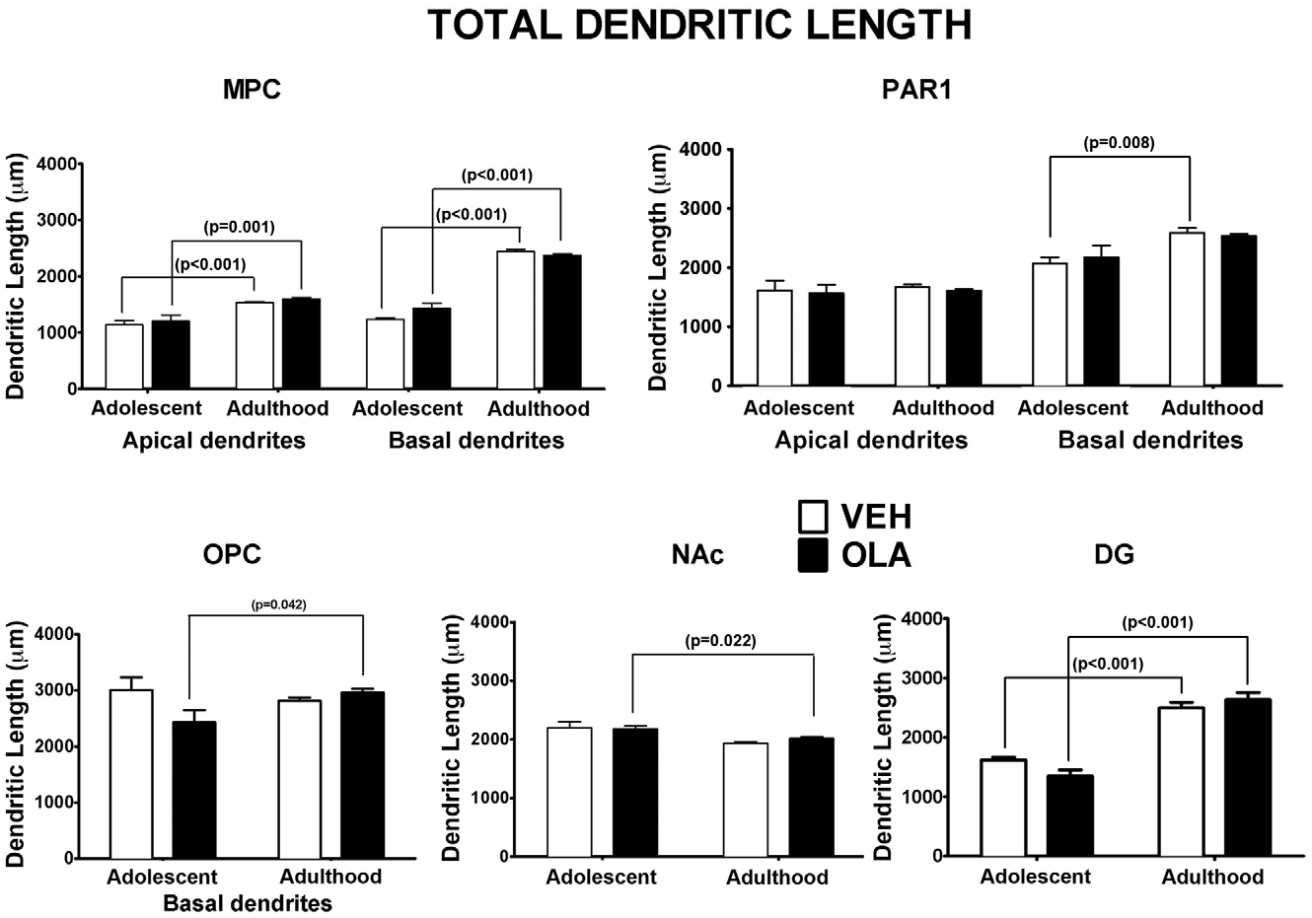
Total dendritic length. There were no statistically significant effects of treatment in adolescents or adults. p-values above brackets indicate the significance of age related changes. All p-values are based on paired t-tests corrected for multiple comparisons. Error bars are standard error of the mean.

The effects of age on the total amount of dendritic arbor are regionally specific (Fig. 7, Table 1). In VEH-treated control rats, total dendritic length increases between P49 and maturity by: 35% and 98% for apical and basal dendrites, respectively, in MPC, 25% for basal dendrites in PAR1 and 54% for granule cells in DG. In the NAc core, there is a trend towards developmental reduction in total dendritic arbor (t[11] = 2.64; p = 0.08), whereas for apical dendrites in PAR1 and basal dendrites in OPC, there were no significant developmental effects.

There are no significant effects of OLA treatment on the total amount of dendritic arbor. However, for basal dendrites in OPC, there is a significant interaction between age and treatment due to the developmental increase in total dendritic length in OLA-treated rats. In NAc, the normal trend towards developmental reduction in dendritic length becomes significant in OLA-treated rats. In PAR1 the normal developmental increase in basal dendritic arbor is blunted and no longer significant.

#### Number and distribution of dendritic segments

**Figure 8:**
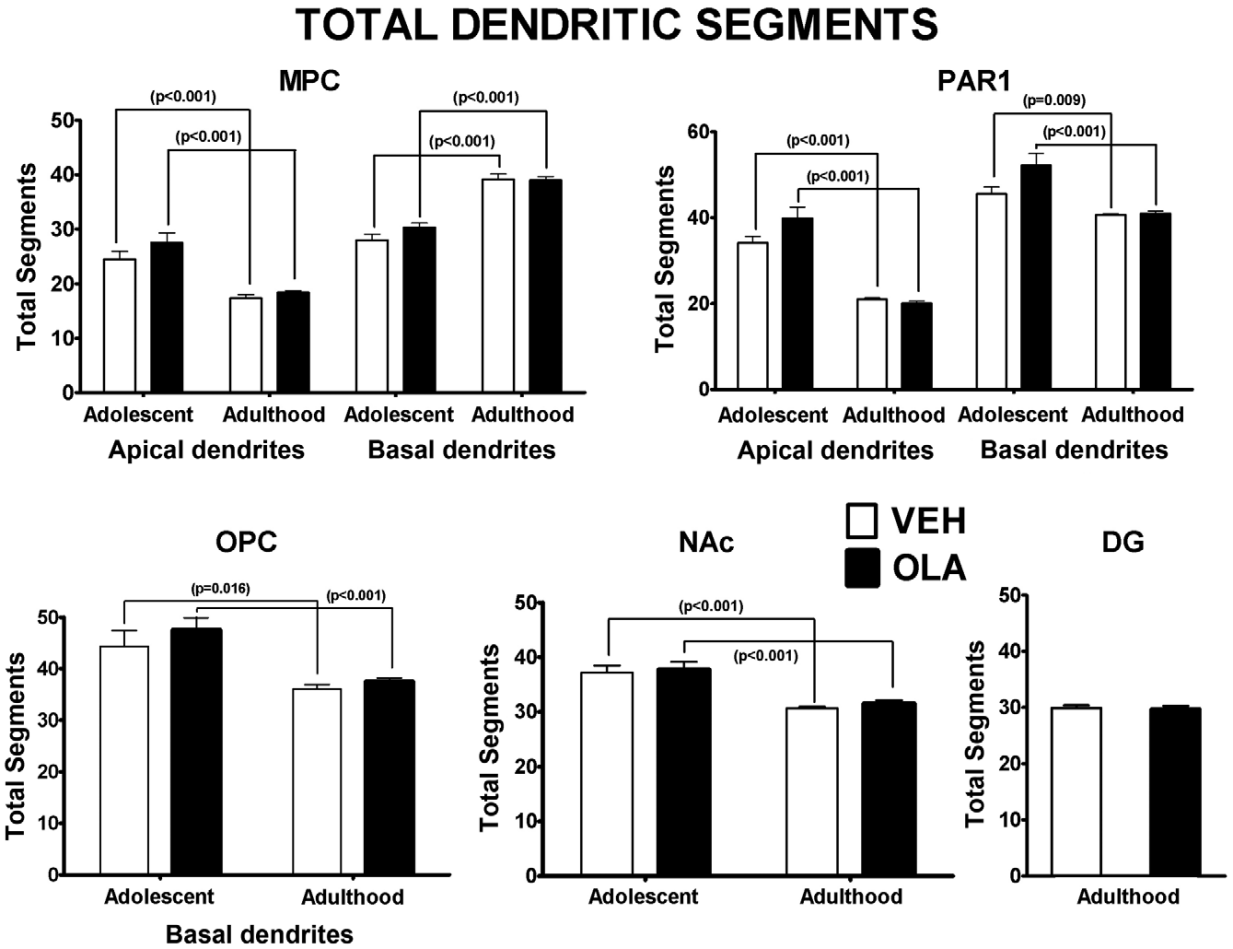
Total number of dendritic segments. There were no statistically significant effects of treatment in adolescents or adults. p-values above brackets indicate the significance of age related changes. All p-values are based on paired t-tests corrected for multiple comparisons. Error bars are standard error of the mean.

There were significant overall effects of age on the total number of dendritic segments for all the dendritic populations analyzed (Table 1). In normal and OLA-treated rats, the total number of dendritic segments decreases between P49 and maturity in all the dendritic populations for which we have data, *except* for basal dendrites in MPC, whose numbers increase (Fig. 8). In PAR 1, there is a tendency towards an increased number of segments in apical and basal dendrites by the end of OLA treatment (P49) and a normalization of segment number at maturity. This is confirmed by the significant interaction between age and OLA treatment for these dendrites. OLA treatment did not significantly alter the distribution of dendritic segments among branches of different order in any of the regions studied (Fig. S5).

### Receptor Binding

**Figure 9:**
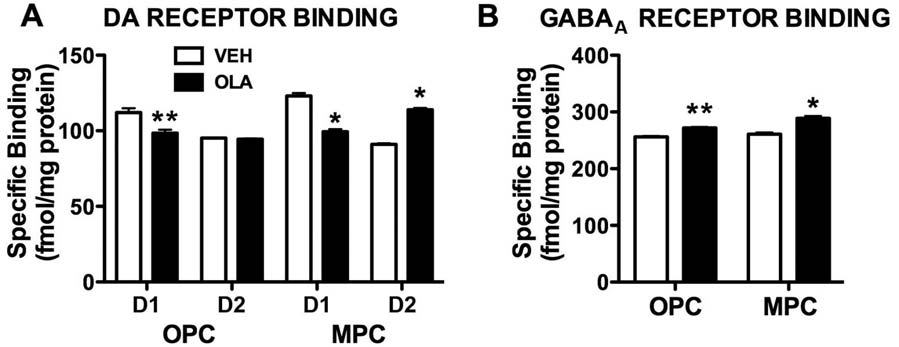
Neurotransmitter receptor binding in the OPC and MPC. A. D1 and D2 receptor binding in OPC and MPC. ** indicates p = 0.022; * indicates p≤0.001. B. GABA_A_ receptor binding in OPC and MPC. ** indicates p = 0.002; * indicates p = 0.005. Error bars are standard error of the mean.

In adult rats (6–8.5 months old) treated with OLA in adolescence, specific D1 binding in OPC and MPC is *decreased* compared to VEH-treated rats (Fig. 9A): in OPC, by ∼12% (VEH = 112.1±2.8, OLA = 98.5±2.2 fmol/mg protein; t[4] = 3.77; p = 0.022) and, in MPC, by ∼19% (VEH = 123±1.9, OLA = 99.4±1.7 fmol/mg protein; t[4] = 9.16; p = 0.001). By contrast, adolescent OLA treatment *increased* specific D2 binding in MPC of adults by ∼25% (Fig. 9A; VEH = 91.1±0.7, OLA = 114.0±1.1 fmol/mg protein; t[4] = 17.95; p<0.001). In OPC, there was no significant OLA-induced change in D2 binding (Fig. 9A; t[4] = 1.15; p = 0.315). We also determined GABA_A_ receptor binding because DA modulates the function of inhibitory interneurons in the prefrontal cortex [45], [46]. Specific GABA_A_ binding in OPC and MPC was *increased* (Fig. 9B): in OPC, by ∼6%, (VEH = 256.2±1.4, OLA = 271.7±1.9 fmol/mg protein; t[4] = 6.76; p = 0.002) and, in MPC, by ∼11%, (VEH = 260.7±3.3, OLA = 289.0±3.9 fmol/mg protein; t[4] = 5.59; p = 0.005).

## Discussion

The principal findings of this study are that adolescent OLA treatment causes: 1) long-term deficits of working memory and extinction of fear conditioning; 2) enduring changes in DAergic receptor binding and GABA_A_ receptor function in OPC and MPC; 3) alterations in the developmental trajectory and mature architecture of the dendrites of layer 3 pyramidal cells in MPC and OPC, and granule cells in DG, although there was no significant effect on layer 3 pyramidal cells in PAR1. These data constitute the first demonstration that adolescent OLA treatment induces highly specific learning deficits that endure long after the termination of treatment and that these behavioral changes are accompanied, in relevant brain regions, by regionally-specific changes in the developmental trajectory and mature configuration of hard-wired neural circuitry and long-term alterations in receptor binding.

### Behavioral changes in response to adolescent OLA treatment

#### Working memory and spatial memory

We found that adult rats treated with OLA as adolescents are impaired in the DNMS working memory task but not in the MWM spatial memory tasks. Rats with MPC lesions made in adulthood (e.g., [47]) are impaired in the acquisition of the DNMS and MWM tasks. We are unaware of any lesion studies in adolescent rats that were trained in these tasks. However, rats with pre-pubertal MPC lesions made on P25 have a chronic deficit in a serial spatial reversal task learned as adults [48], although it is less than that of rats with similar lesions made in adulthood. Thus, our finding that OLA treatment begun pre-pubertally induces chronic working memory impairment is consistent with MPC lesion data.

Why is DNMS performance, but not MWM performance, impaired? The DNMS task requires continuous monitoring of the most recent information trial, thus taxing working memory. As noted by Pribram [49] in his analysis of delayed response deficits in monkeys with dorsolateral prefrontal injury, one of the requirements of the task is not only to keep track of the information signal but also to recall *only the most recent signal*. The MWM tasks are very different: the rats must develop a strategy that includes 1) leaving the pool wall to seek a way out of the water and 2) forming and using a spatial map based on room cues. MPC is believed to be involved in the former requirement but once the strategy to solve the task is formed, MPC likely has little role in the performance of the task. This can be seen, for example, in the absence of a retention deficit in MPC lesion rats pre-trained on the Morris task [50]. The moving platform MWM task clearly has a working memory component after the initial daily information trial, but in contrast to the DNMS task, the correct choice is constant for all trials that day. Thus, given the expectation of a reduced deficit after adolescent perturbation of MPC, it would seem that only more difficult tasks, such as the DNMS task used here, will reveal impairment following adolescent OLA treatment.

#### Acquisition/extinction of fear conditioning

Although adult rats treated with OLA as adolescents exhibit normal acquisition of fear conditioning, they appear to be hyper-responsive: extinction of conditioned fear to CS presentation is impaired and, post-conditioning, freezing to the context of conditioning is enhanced. Also, on Day 1, prior to conditioning, OLA-treated rats freeze more than controls in response to presentation of the CS. These effects may reflect functional alteration of the MPC [51], [52], perhaps due to the OLA-induced changes in D1-, D2- and GABA_A_ receptor binding, or the changes in dendritic spine density that we observed. All of these changes are likely to alter the balance of excitatory and inhibitory drive converging on prefrontal cortical neurons, and thus, the properties of the prefrontal cortical network. Infralimbic MPC lesions impair aspects of extinction, but do not affect acquisition, of fear conditioning [53], [54]. By contrast, prelimbic MPC lesions promote fear responses to cue and context during both acquisition and extinction [51]. On Day 1, the relatively strong shock used in our study may have produced a ceiling effect that caused conditioned freezing to the cue in VEH-treated rats to reach the same level as in OLA-treated rats, even though there was no ceiling effect for context conditioning, which was stronger in OLA-treated rats (as assessed on Day 2). The relatively strong shock in our study may explain why extinction was not more pronounced in our VEH-treated control rats.

The MPC projects to the basolateral amygdala complex (BLA) [52], [55], which is critical for fear responses [56]. Stimulation of the MPC inhibits fear responses [57], in part by activating inhibitory interneurons in the BLA [52], [58]. Activation of DA receptors attenuates PFC input to the BLA and likely increases behavioral responding to aversive stimuli [58], [59], [60]. Thus, the increased freezing of OLA-treated rats described above could be due to reduced tone of MPC input to the BLA (secondary to re-organization of the MPC) or to altered DAergic function in the BLA. Interestingly, individuals diagnosed with post-traumatic stress disorder (PTSD) exhibit impaired extinction to a CS previously associated with aversive stimuli [61]. This deficit appears to depend on brain regions homologous to those that mediate extinction of fear conditioning in rats [62]. Thus, adolescent OLA treatment may increase the likelihood of developing anxiety-related disorders like PTSD.

The impaired extinction of fear conditioning following adolescent OLA treatment appears not to be related to general alterations in motor activity, as OLA- and VEH-treated animals did not differ significantly with respect to the amount of freezing during the first 120 s after being placed in the test chamber on Day 1 (Fig. 4C), or their locomotor activity in a novel open field (Fig. S1). Similarly, the effects of OLA on fear conditioning are not due to a chronic, non-specific increase in fear, as OLA- and VEH-treated rats do not differ significantly in their elevated plus maze behavior (Fig. S3).

#### Specificity of the sequelae of adolescent OLA treatment

Our adult rats treated with OLA as adolescents were impaired in working memory and the extinction of fear conditioning, but not in spatial learning or the acquisition of fear conditioning. Thus, the learning deficits caused by adolescent OLA treatment are highly specific and not due to perturbation of mechanisms on which all forms of learning are dependent. Performance on other tasks is [32], or may in the future be found to be, altered. However, the behavioral sparing in our open field, motor skill and elevated plus maze tasks, and the absence of any significant effects on the CORT response to transient stress, demonstrate that the long-term effects of adolescent OLA treatment are restricted to specific behaviors and their underlying neural networks.

#### Comparison with other studies

Our behavioral results are congruent with those of others [28], who assayed the effects of OLA treatment on P28–49 slightly later in adolescence (P60–71), rather than in mature animals (current results). These investigators found a significant deficit in a working memory task (novel object recognition) and no significant effects on measures of anxiety. Comparison of our data with those of other studies is complicated by differences in the drugs studied and, in one case, species. The spectrum of receptors engaged by OLA (and all other atypical APDs) is highly dose-dependent. At very low doses, atypical APDs antagonize principally 5HT2A receptors; at higher doses, they antagonize D2, D4 and 5HT2C receptors, and others, notably muscarinic ACh receptors, α-adrenergic receptors and H1 histamine receptors [3], [63]. As noted in Methods, the dose of OLA we used was chosen to produce D2 receptor occupancies in the human therapeutic range.[33], [34] In contrast to our experiment, several other studies (reviewed in [64]) investigated the effects of treating adolescent rats with two other atypical APDs, risperidone or clozapine, at low doses for which the principal action of the drugs is 5HT2A receptor antagonism. D2 occupancy would have been far below the human therapeutic range at peak and undetectable for much of each day.[33], [34] In rats subjected to neonatal ventral hippocampal lesions or fetally exposed to a maternal inflammatory response caused by poly I:C treatment, the adolescent low dose APD administration blocked the induction of behavioral phenotypes of schizophrenia that, without further intervention, typically emerge by adulthood. In control rats, the adolescent APD treatments had no adverse effects when the rats were tested as adults on the same tasks. Together, these results and our data suggest three, translationally important hypotheses for future testing: 1) In contrast to adults, in adolescents, atypical APDs can exert their therapeutic effects when the ratio of D2 antagonism to 5HT2A antagonism is very low; 2) the deleterious effects of adolescent atypical APD administration depend, to a large extent, on either D2 receptor signaling alone or an elevated ratio of D2/5HT2A antagonism; 3) APD treatment of otherwise healthy adolescent rats may produce adverse, long term behavioral and neurobiological effects that are absent when the drugs are administered at the same dose. The body of available data has two important lessons for clinicians: 1) Atypical antipsychotic therapy may require different strategies in adolescents and adults; 2) although preventive APD therapy may be beneficial in some individuals with an elevated risk for psychosis-related behavioral dysfunction, prophylactic treatment of patients who might not otherwise develop psychotic symptoms, may carry substantial risk of long-term behavioral deficits.

#### Adolescent vs adult OLA treatment

We are unaware of behavioral studies of adult rats conducted after an extended period of withdrawal from chronic APD treatment. Adult rats studied at various intervals *during* chronic OLA treatment at doses similar to ours exhibit short term memory deficits (15 min–6 h delay), impaired acquisition and retention of spatial learning in the MWM and motor deficits [65]. Although our results on the long-term effects of adolescent OLA treatment stand by themselves, future experiments in which the behavioral and neurobiological sequelae of equivalent adolescent and adult treatments are compared at similar post-treatment intervals will be required to determine which effects are specific to the age at which OLA is administered and whether effects common to adolescent and adult treatment have the same underlying mechanisms at both ages.

### Dendritic Architecture

The amount, branching complexity and spatial distribution of dendritic arbor, and dendritic spine density, are major determinants of neuronal connectivity and dendritic signal integration [66], [67]. Thus, alterations of dendritic form are indicators of changes in the “hard wiring” and functional properties of neuronal networks, which can profoundly impact behavior. In this study, measures made on P49 (the last day of OLA treatment) reveal the effects of OLA while the drug was present, whereas measures obtained at 8 months of age reflect any subsequent maturational changes, and thus, the status of the brain during the period of behavioral testing and prior to changes associated with advanced age.

#### Dendritic architecture in mature rats

Spiny neurons receive the preponderance of their excitatory input on dendritic spines [67]. Therefore, OLA-induced changes in local spine density indicate parallel alterations in the amount of excitatory input converging on the corresponding dendritic region [67]. In our *mature* rats treated with OLA as adolescents, dendritic spine density is significantly elevated in MPC and OPC, reduced in DG and not significantly altered in PAR1 or the NAc (Fig. 6). There is a similar regional specificity for the effects of pre-pubertal amphetamine treatment on dendritic spine density (c.f., [68], [69]). This specificity could be due to regional differences in the relative weighting of diverse signaling mechanisms that modulate dendritic spine density, or to regional variations in the schedule of dendritic development, that result in distinct “critical periods” during which early life OLA treatment can alter spine density long term. There are no significant long-term effects of OLA on total dendritic length (Fig. 7) or on branching complexity (Fig. 8). These data contrast with the robust effects (usually decreases) on *all 3* measures of dendritic form in adult mice treated *pre-weaning* with OLA on P3–10 [70]. The most likely explanation for this is that within all ROIs, by adolescence, dendritic length and branching complexity are no longer sensitive to APD treatment, whereas dendritic spine density in MPC, OPC and DG still is.

#### Dynamics of dendritic development

Adolescent OLA treatment has even more varied effects on the *dynamics* of dendritic maturation. The normal development of dendrites, spines and synapses involves both selective growth and regressive (“pruning”) processes that can occur simultaneously in the same dendritic arbor [24], [71], [72], [73], [74], [75], [76], [77], [78]. In VEH-treated control rats, *dendritic spine density* decreased significantly between P49 and adulthood in all 5 ROIs (Fig. 6, Table 1). This is consistent with prior findings that in the primate cerebral cortex, synapses develop “concurrently and at identical rates” in different layers and in all cortical areas, and the suggestion that across the cerebral cortex, synaptic development may be orchestrated by a common signal [72].

Adolescent OLA treatment alters the *dynamics* of dendritic maturation in a regionally specific manner, *even after the cessation of treatment* on P49 (Fig. 6, Table 1). In MPC of OLA-treated rats, *spine density* is the same at P49 and in adulthood, although it normally decreases during that interval. Our data do not indicate whether this effect is due to a temporal shift of the normal schedule of spine proliferation and pruning or to a subnormal proliferation of spines that is stabilized once it reaches its maximum (by P49). Either way, the altered dynamics of dendritic spine ontogeny resulting from adolescent OLA treatment suggests the disruption of activity-dependent developmental processes in affected brain regions as a neurobiological mechanism of long-term network- and behavioral dysfunction. In OPC of OLA-treated rats, spine density increases between P49 and adulthood, in contrast to the decrease in VEH-treated control rats. Altered developmental dynamics of dendritic spines and synapses may be a common response to early life environmental and pharmacologic challenges, although it is likely to depend on the type of challenge and to be region- and developmental stage-specific [76], [79]. For example, pre-weaning stress, like OLA, has a delayed effect on the dynamics of synapse maturation in areas CA1 and CA3 of the hippocampus, but not in the amygdala [76]. Similarly, prenatal exposure to another psychoactive drug, nicotine, also causes regionally specific changes in the dynamics of maturation in dendritic spine density in the MPC, OPC and NAc during juvenile and adolescent stages (c.f., [80], [81]). Dendritic architecture and patterns of neuronal activity can reciprocally modulate each other [78]. Thus, the altered dynamics of dendritic spine ontogeny resulting from adolescent OLA treatment suggests the disruption of activity-dependent developmental processes in affected brain regions as a neurobiological mechanism of network- and behavioral dysfunction.

The developmental changes in *total dendritic length* are regionally specific in VEH-treated, control rats (Fig. 7, Table 1). In PAR1, total dendritic length increases between P49 and adulthood in basal-, but not apical dendrites. Thus, in some regions, normal developmental changes in the total length of dendrites originating from the same cell population can be layer-specific. Although OLA treatment has no main effects on total dendritic length, it alters the dynamics of growth in OPC so that in mature rats, total dendritic length is greater than on P49. In NAc, the normal trend towards developmental reduction in dendritic length becomes significant in OLA-treated rats.

The normal developmental trajectory of *dendritic branching complexity* (number of dendritic segments; Fig. 8, Table 1) is both regionally- and layer specific. The total number of dendritic segments decreases between P49 and maturity in all the dendritic populations for which we have data, *except* for basal dendrites in MPC, whose numbers increase. OLA treatment appears to have little effect on dendritic branching complexity other than a minor alteration of the dynamics of its maturation in PAR1. The absence of significant effects of OLA treatment on the distribution of dendritic segments among axon branches of different order (Fig. S5) indicates that there is no significant redistribution of dendritic segments by branching order as a result of OLA treatment, aging, or their interaction.

Together, our Golgi data show that adolescent OLA treatment alters, in a regionally specific fashion, the dynamics and/or final outcome of ontogenetic changes in dendritic spine density in MPC, OPC, and DG and the total amount of dendritic arbor in OPC, but has virtually no influence on dendritic branching complexity. Importantly, many of the effects on spine density occur after the completion of OLA treatment and persist well into adulthood, perhaps even over the entire life span. These data identify dendritic spine density - a measure of the density of excitatory input converging on individual neurons [67] - as a key circuit feature of the OPC, MPC and HIPP targeted by adolescent OLA treatment and a likely contributor to changes in behaviors mediated by these brain regions.

### Receptor Binding

Our receptor binding data suggest 3 potential, mutually compatible mechanisms of the behavioral and neurobiological sequelae of adolescent OLA treatment:

#### Altered neurotransmitter receptor signaling in the mature prefrontal cortex can perturb cortical network function and behavior

In the prefrontal cortex, D1 and D2 receptors are present on both pyramidal cells and GABAergic interneurons starting prior to puberty [36], [82]. In the mature MPC, D1 receptors enhance the excitability of layer 5/6 pyramidal cells [83], [84], whereas D2 receptors attenuate pyramidal cell excitability [46], [83]. The D2 effects are due to a direct action on pyramidal neurons and to the activation of GABAergic fast-spiking (FS) interneurons. [83]. Thus, the changes in D1- and D2 binding in adult rats treated with OLA as adolescents are likely to alter the excitability of pyramidal cells and interneurons, and the properties of prefrontal cortical networks. In humans [85] and animal models [83] DAergic signaling is critical in optimizing the signal-to-noise ratio of prefrontal cortical activity during the performance of working memory tasks. The phasic release of DA and its activation of D1 receptors in the prefrontal cortex are critical for working memory function [86]. Thus, the long-term reduction in D1 binding induced by adolescent OLA treatment is a likely mechanism of the working memory deficit that occurs when the rats are adults. The deficit in extinction of fear conditioning caused by adolescent OLA treatment may be due to a similar reduction in D1 function in the inferior MPC [87]. Increased prefrontal GABA_A_ receptor binding (this report) and altered 5HTergic function [64] are additional likely mediators of these behavioral effects.

#### OLA- induced changes in the ontogenetic trajectory of receptor binding can alter the functional development of neuronal networks

In the prefrontal cortex of normal rats, D1 and D2 binding increase rapidly from P28–40, then decline to mature values from P60 to P100 [35], [36], [37]. *Acute* APD treatment antagonizes D2 receptors. However, in mature animals [29], [88], [89], [90] and human patients [91], [92], *chronic* treatment with typical- and atypical APDs (including OLA) over a protracted period can up-regulate D2 binding, depending on the duration and percentage of D2 receptor blockade. This causes a loss of therapeutic efficacy in patients [91], [92]. D2 binding returns to normal levels within a few weeks following withdrawal of treatment, with [88], [90] or without [93] D2 up-regulation. By contrast, we show here that, months after the cessation of adolescent OLA treatment on P28–49, D2 binding is increased in MPC, whereas D1 binding in MPC and OPC is decreased. This indicates an altered developmental trajectory of receptor binding. The 5HTergic activities of OLA raise the possibility that the ontogenetic trajectories of some 5HT receptors may also be altered by adolescent OLA treatment. The mechanistic contribution of OLA-induced changes in D1, D2 (and, possibly, 5HT) receptors to alterations of behavior and neuronal circuitry will require additional studies of receptor binding and function at multiple developmental time points. Early life stimulant exposure, like adolescent OLA exposure, can induce enduring changes in D1- and D2 family receptor expression [94], [95], [96] and function [94], [96].

Importantly, in the MPC, the excitability-enhancing effect of D1 receptors [84] and the recruitment of GABAergic interneurons by D2 activation [45], [46] emerge during adolescence. Thus, OLA-induced changes in the developmental sequence of D1 and D2 binding are likely to alter the ontogenetic trajectories of excitatory- and inhibitory tone in the prefrontal cortex and to perturb activity-dependent developmental processes. This provides an additional potential mechanism for behaviorally significant alterations in cortical network function [82]. Our observations on GABA_A_ binding support this possibility. EBOB is a non-competitive GABA_A_ antagonist whose binding to the GABA_A_ allosteric site is enhanced when the Cl^−^ channel of the receptor is in the open configuration [97]. Thus, the increase in EBOB binding in OPC and MPC of adult rats treated with OLA as adolescents suggests an elevation of inhibitory tone in those regions, due to increases in the number of binding sites and/or the percentage of open Cl^−^ channels. We are beginning neurophysiological investigations of this mechanistically important hypothesis.

#### OLA- induced changes in the ontogenetic trajectory of receptor binding can alter the development of neuronal connectivity

In many brain regions, normal ontogenetic changes in dendritic architecture and other features of neuronal connectivity are regulated by the level and pattern of electrical activity [78], [98], [99] and by monoaminergic signaling [21], [100]. Thus, early life exposure to therapeutic drugs [70], [101], [102] and stimulants [21], [68], [103], [104], [105], [106], [107] that modify activity or monoamine signaling can alter dendritic development. The effects of stimulants on dendritic form and spine density occur preferentially in brain regions with robust DAergic and 5HTergic input [104], [108], [109], [110] input and can be mediated by D1- [108], [111], [112] and D2 [112] receptors. These observations invite future investigations to determine if OLA-induced changes in the developmental trajectory of DA- (and, potentially, 5HT- and GABA_A_-) receptor binding impact behavior by altering dendritic form and, therefore, cortical network properties.

## Conclusion

Adult rats treated with OLA as adolescents exhibit specific deficits in working memory and extinction of fear conditioning (current data), as well as enhanced conditioned place preference [32]. These behavioral changes are accompanied by long-term alterations in DAergic- and/or GABAergic signaling in MPC, OPC, (and NAc [32]) and by modification of the maturational dynamics and mature levels of dendritic spine density and/or amount of dendritic arbor in those regions and in DG. Altered DAergic and GABAergic transmission are likely mediators of these effects, although other transmitter systems may also be involved. Our findings raise the possibility of additional, long-term impacts on behaviors mediated by MPC, OPC or NAc function and of alterations in the network properties in those regions. The persistence of the preceding changes emphasizes the importance of weighing the benefits and risks of adolescent APD therapy, especially prophylactic treatment in high risk, asymptomatic patients. Our data also suggest that adolescent APD treatment may also cause enduring changes in behavioral- and neurobiological responses to other therapeutic- or illicit psychotropic drugs.

## Supporting Information

Figure S1 **Open field exploration.** For 30 min on each of 4 consecutive days, rats were placed in a plexiglass arena (42 cm×42 cm; 30 cm walls) and their locomotor exploratory behavior was monitored using an infrared beam break system. Exploratory activity was measured by the number of horizontal beam breaks per 5 min bin. Data were analyzed by 3-way ANOVA: within subjects factors were time in session (5 min bins, to assay within-session habituation) and day of session (1–4, to assay between-session habituation); the between subjects factor was treatment (VEH/OLA).Frames A–D show the number of horizontal beam breaks (a measure of distance traveled) in an open field on each of 4 consecutive days of testing. The data were broken into 5 min bins. The inset in frame D shows the total number of beam breaks in each of the 4 sessions, which permits visualization of activity changes that occur across test sessions. Error bars are standard error of the mean.Activity decreased significantly across sessions (Fig. S1; F[3,54] = 7.67, p<0.001) and within sessions (Fig. S1; F(5,90) = 89.3, p<0.001). This indicates habituation to a novel environment. OLA- and VEH-treated rats did not differ significantly in overall activity pooled over the 4 sessions (F[1,18] = 0.053 p = 0.82). The treatment X session, and treatment X time bin interactions also were not significant (F[3,18] = 0.59, p = 0.62, and F[5,18] = 0.61, p = 0.69, respectively). Thus, adolescent OLA treatment does not cause any significant change in overall motor responsiveness to a novel environment or any gross motor deficit.
(TIF)

Figure S2 **Skilled reaching.** A. Tray reaching**.** This procedure [113] was used to assess the skilled forelimb movements of each rat after training to reach for chicken feed pellets. Rats were placed in Plexiglas cages (28 cm deep ×20 cm wide ×25 cm high), the front and floor of which were constructed of 2 mm bars separated by 1 cm, edge to edge. A tray (5 cm deep ×2 cm wide ×1 cm high) containing the pellets, was mounted in front of each cage. To obtain pellets, the rats had to extend a forelimb through the bars, grasp, and retract the food pellet. The food tray was mounted on runners to adjust the distance of the food from the bars. Distance adjustments ensured that each rat could not simply take the food into the cage. Bars on the floor ensured that a dropped the pellet would be irretrievable and the rat would have to reach again. Rats were trained on the task for a maximum of three weeks before video recording. During the first week, the rats were grouped in pairs in the reaching cages for one h/d to allow them to adapt to the apparatus. Food restriction also began during the first week; each rat received 15 g/d of laboratory chow following the training period. Weight was monitored to ensure that the rats' weight did not fall below 90% *ad libitum* feeding values. The rats were subsequently trained individually for 1 h/d during the second week; during the third week, this was shortened to 5–15 min/d. Five min of continuous reaching activity for each rat was videotaped and scored when the rats were ∼5 months old. Insertion of a forepaw through the bars, without grasping food or dropping the food, was scored as a "reach”. If the rat obtained a piece of food and consumed it, the movement was scored as a “reach” and a “hit.” We calculated the percentage of hits/total reaches for each rat's preferred forelimb. All rats learned the task and there were no significant OLA-induced changes in the number of reaching attempts (not illustrated; VEH = 115.9±28.9, OLA = 89.5±16.2; F[1,18] = 0.7, p = .415) or in the average percentage of successful reaches (VEH = 45.8±10.5; OLA = 60±4.8; F[1,18] = 1.7, p = .21). Error bars are standard error of the mean.B. Single Pellet Reaching. This procedure [114] is designed for studying effects on individual components of a reaching movement. The reaching box was made of clear Plexiglas (45 cm long ×14 cm wide ×35 cm high. In the middle of the front wall, a 1 cm wide slit extended from the bottom of the box to a height of 15 cm. A 2 cm wide by 4 cm long shelf was attached to the outside of the front wall, 3 cm above the bottom of the box in front of the opening. There were two small indentations 1.5 cm from the front wall aligned with each side of the slot to hold the food pellets. Food pellets in each indentation are accessible only to the contralateral hand, because rats can only pronate medially to grasp the food. The rats were trained to walk to the back of the box to retrieve a loose food pellet and then to walk to the slot and reach for a pellet. Initially, pellets were presented in both indentations. Once a rat showed a clear preference for one side, the food pellets were placed only in the indentation accessible to the preferred forelimb. Once the rats had learned the task, which took ∼7 days, they were tested for 7 days with 25 trials/day. On the last day, the first 5 trials were videotaped for kinematic analysis. The kinematic analysis consisted of rating 10 distinct reaching elements [114]. Performance of each element was rated on a three-point scale to obtain movement scores. A score of “0” was given for a normal movement, a score of “0.5” was given for abnormal movement, and a score of “1” was given for the absence of the movement. In the present analysis, we used the sum of the ratings on all 10 components to score each reaching movement (i.e., each trial could receive a score of 0–10). All rats learned the task. There were no significant treatment effects on mean success rate over the 7 days of testing (VEH = 51.8±3.7%; OLA = 51.7±2.4%; F[1,18]<0.001, p = .99) or on mean total element scores obtained in the kinematic analysis (not illustrated; VEH = 0.15±.3; OLA = 0.21±.32; F[1,18] = 1.7, p = .21). Error bars are standard error of the mean.
(TIF)

Figure S3 **Elevated plus maze.** The EPM, a “+” shaped maze with 2 closed- and 2 open arms, was used to test anxiety-like behavior. Each arm measured 113×10 cm. The maze was 88 cm above the floor. Rats were placed in the center of the maze facing a closed arm and were allowed to explore freely for 5 min. Time spent in the open arms is an inverse measure of an anxiety-like phenotype in rodents. There was no significant effect of treatment (T[1,18] = 0.664; p = 0.426). Error bars are standard error of the mean.
(TIF)

Figure S4 **Hypothalamo-pituitary-adrenal (HPA) axis responses to mild, transient stress.** All test procedures were initiated starting ≥1.5 h after lights on and were completed no later than 5.5 h after lights on, to guarantee that all measurements were made during the phase of the diurnal cycle when ACTH and corticosterone (CORT) levels are constant and at their nadir. For a week prior to testing, subjects were brought daily to the procedure room, to habituate them to being moved from the adjacent colony room. On the first day of testing, baseline blood samples were obtained by tail nick immediately after subjects were brought into the procedure room. After a 72 h recovery period, subjects were placed in a plastic restrainer immediately after being brought into the procedure room; restraint was maintained for the first 20 min of the 2 h test; for the remainder, subjects were placed in a holding cage, after which they were returned to their home cages. Blood samples were obtained by tail nick at 15, 30, 60 and 120 minutes following the onset of restraint. Samples (∼300 µl) were typically obtained ≤1 min (always ≤2 min) post-nick. Blood was collected in EDTA-coated tubes and maintained at 4°C during testing. At the completion of testing, blood samples were centrifuged at 2000×g for 10 min at 4°C and the plasma was aliquoted into Eppendorf tubes and stored at −80° C. Measurements of plasma CORT were obtained in duplicate using I^125^ radioimmunoassay performed at the University of Virginia Center for Research in Reproduction Ligand Assay and Analysis Core Laboratory (http://www.medicine.virginia.edu/research/institutes-and-program/crr/lab-facilities). The effects of treatment, time and their interaction were determined by repeated measures ANOVA.There was no significant treatment effect on baseline plasma CORT concentration (not illustrated; t[22] = 1.038; p>0.05; OLA = 114.1±17.3 ng/mL; VEH = 142.9±22.2 ng/mL). Plasma CORT concentration (baseline subtracted) showed a significant overall effect of time following onset of restraint (F[3,66] = 30.745, p<0.001) and peaked at 60 min. There was no significant main effect of treatment (F[1,22] = 0.003; p>0.05) or treatment X time interaction (F[3,66] = 0.774; p>0.05). There was also no effect of adolescent treatment on the integrated stress response (area under the curve; data not shown; t(22) = 0.817; p>0.05).**Plasma CORT response to 20 min restraint stress.** All values shown on the vertical axis have the baseline value subtracted. The bar underneath the X-axis indicates the interval of restraint. Error bars indicate standard error of the mean.
(TIF)

Figure S5 **Distribution of dendritic segments among branches of different order.** We made a supplemental analysis of our data on the number of dendritic segments of each order because changes in the distribution of branching among segments of different order, which would not be detected by analysis of the total number of branches, can affect the integration of inputs converging on the dendritic tree. The curves at the bottom of each frame show the number of segments of each branch order for the ROI and age indicated. The cumulative distributions in the insets show the percentage of branches at or below each branch order. Error bars are standard error of the mean. Two-way repeated measures ANOVA of the number of segments of each order did not reveal any significant effects of treatment or branch order X treatment interaction.
(TIF)
