# Supplementary figures and images for: Olanzapine Treatment of Adolescent Rats Causes Enduring Specific Memory Impairments and Alters Cortical Development and Function
Source: PLoS One. 2013 Feb 20;8(2):e57308. doi: 10.1371/journal.pone.0057308 (PMC3577739; doi:10.1371/journal.pone.0057308)

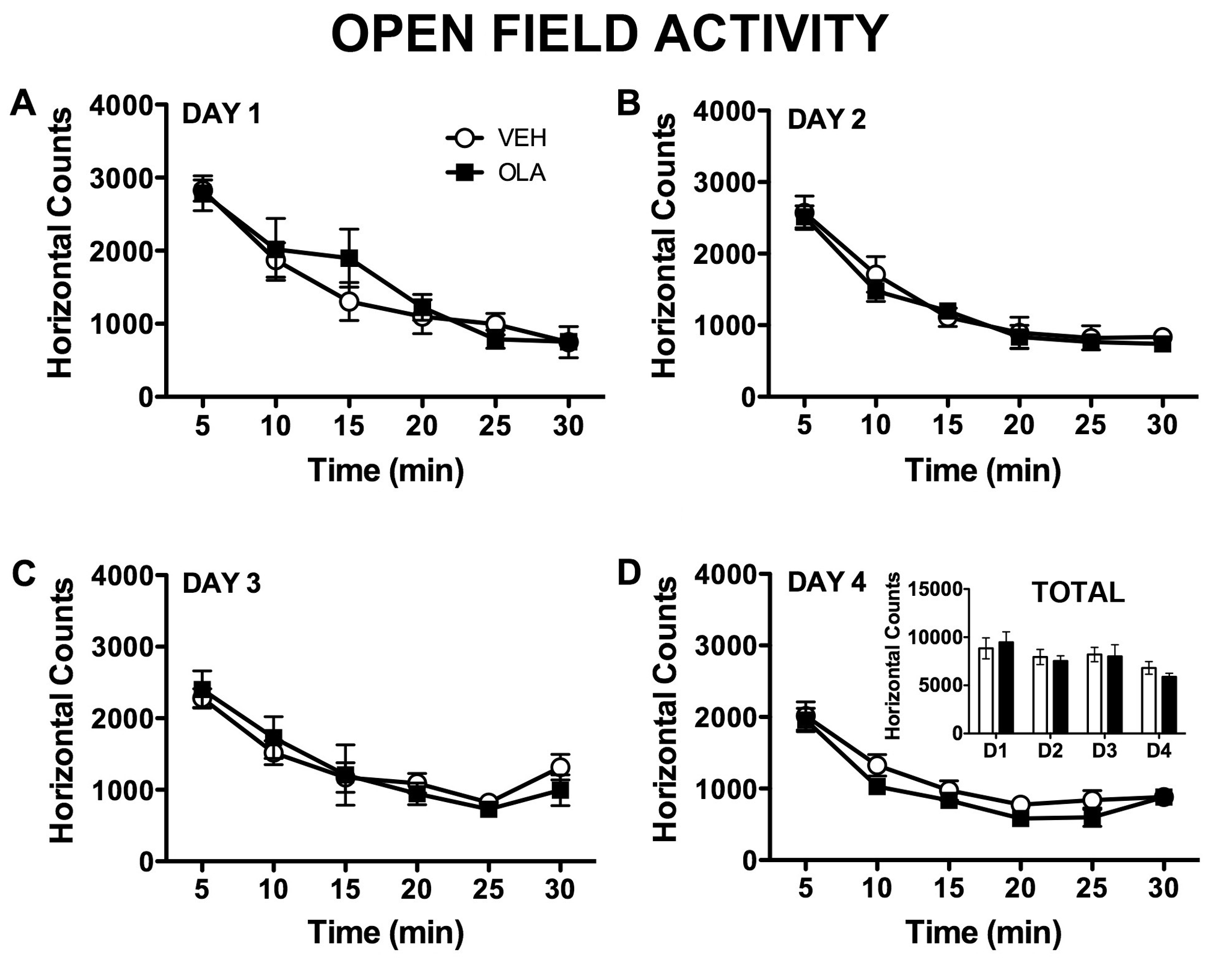

Supplement: Figure S1 — Open field exploration. For 30 min on each of 4 consecutive days, rats were placed in a plexiglass arena (42 cm×42 cm; 30 cm walls) and their locomotor exploratory behavior was monitored using an infrared beam break system. Exploratory activity was measured by the number of horizontal beam breaks per 5 min bin. Data were analyzed by 3-way ANOVA: within subjects factors were time in session (5 min bins, to assay within-session habituation) and day of session (1–4, to assay between-session habituation); the between subjects factor was treatment (VEH/OLA).Frames A–D show the number of horizontal beam breaks (a measure of distance traveled) in an open field on each of 4 consecutive days of testing. The data were broken into 5 min bins. The inset in frame D shows the total number of beam breaks in each of the 4 sessions, which permits visualization of activity changes that occur across test sessions. Error bars are standard error of the mean.Activity decreased significantly across sessions (Fig. S1; F[3,54] = 7.67, p<0.001) and within sessions (Fig. S1; F(5,90) = 89.3, p<0.001). This indicates habituation to a novel environment. OLA- and VEH-treated rats did not differ significantly in overall activity pooled over the 4 sessions (F[1,18] = 0.053 p = 0.82). The treatment X session, and treatment X time bin interactions also were not significant (F[3,18] = 0.59, p = 0.62, and F[5,18] = 0.61, p = 0.69, respectively). Thus, adolescent OLA treatment does not cause any significant change in overall motor responsiveness to a novel environment or any gross motor deficit. (TIF) [file pone.0057308.s001.tif]

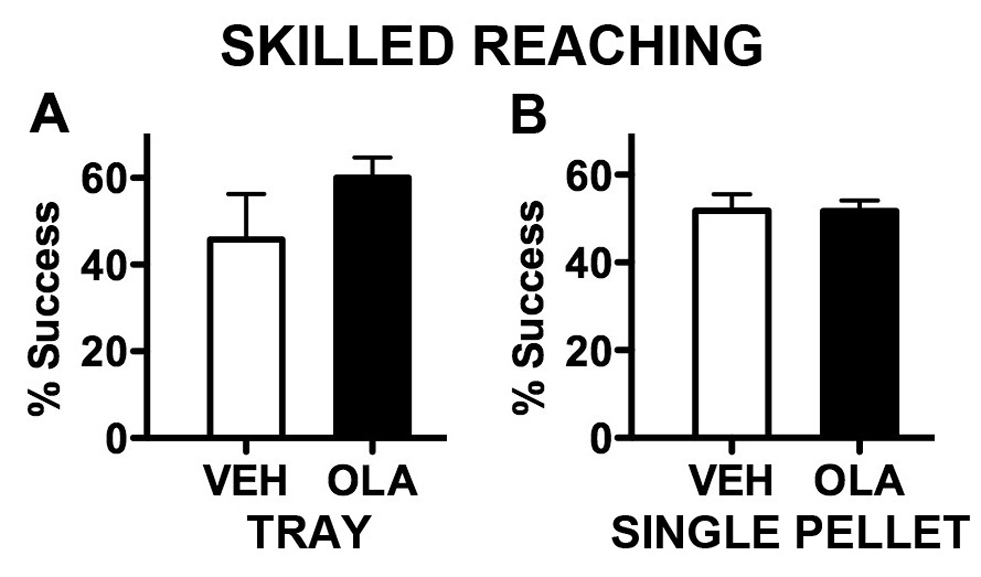

Supplement: Figure S2 — Skilled reaching. A. Tray reaching. This procedure [113] was used to assess the skilled forelimb movements of each rat after training to reach for chicken feed pellets. Rats were placed in Plexiglas cages (28 cm deep ×20 cm wide ×25 cm high), the front and floor of which were constructed of 2 mm bars separated by 1 cm, edge to edge. A tray (5 cm deep ×2 cm wide ×1 cm high) containing the pellets, was mounted in front of each cage. To obtain pellets, the rats had to extend a forelimb through the bars, grasp, and retract the food pellet. The food tray was mounted on runners to adjust the distance of the food from the bars. Distance adjustments ensured that each rat could not simply take the food into the cage. Bars on the floor ensured that a dropped the pellet would be irretrievable and the rat would have to reach again. Rats were trained on the task for a maximum of three weeks before video recording. During the first week, the rats were grouped in pairs in the reaching cages for one h/d to allow them to adapt to the apparatus. Food restriction also began during the first week; each rat received 15 g/d of laboratory chow following the training period. Weight was monitored to ensure that the rats' weight did not fall below 90% ad libitum feeding values. The rats were subsequently trained individually for 1 h/d during the second week; during the third week, this was shortened to 5–15 min/d. Five min of continuous reaching activity for each rat was videotaped and scored when the rats were ∼5 months old. Insertion of a forepaw through the bars, without grasping food or dropping the food, was scored as a "reach”. If the rat obtained a piece of food and consumed it, the movement was scored as a “reach” and a “hit.” We calculated the percentage of hits/total reaches for each rat's preferred forelimb. All rats learned the task and there were no significant OLA-induced changes in the number of reaching attempts (not illustrated; VEH = 115.9±28.9, OLA = 89.5±16.2; F[1,18] = 0 [file pone.0057308.s002.tif]

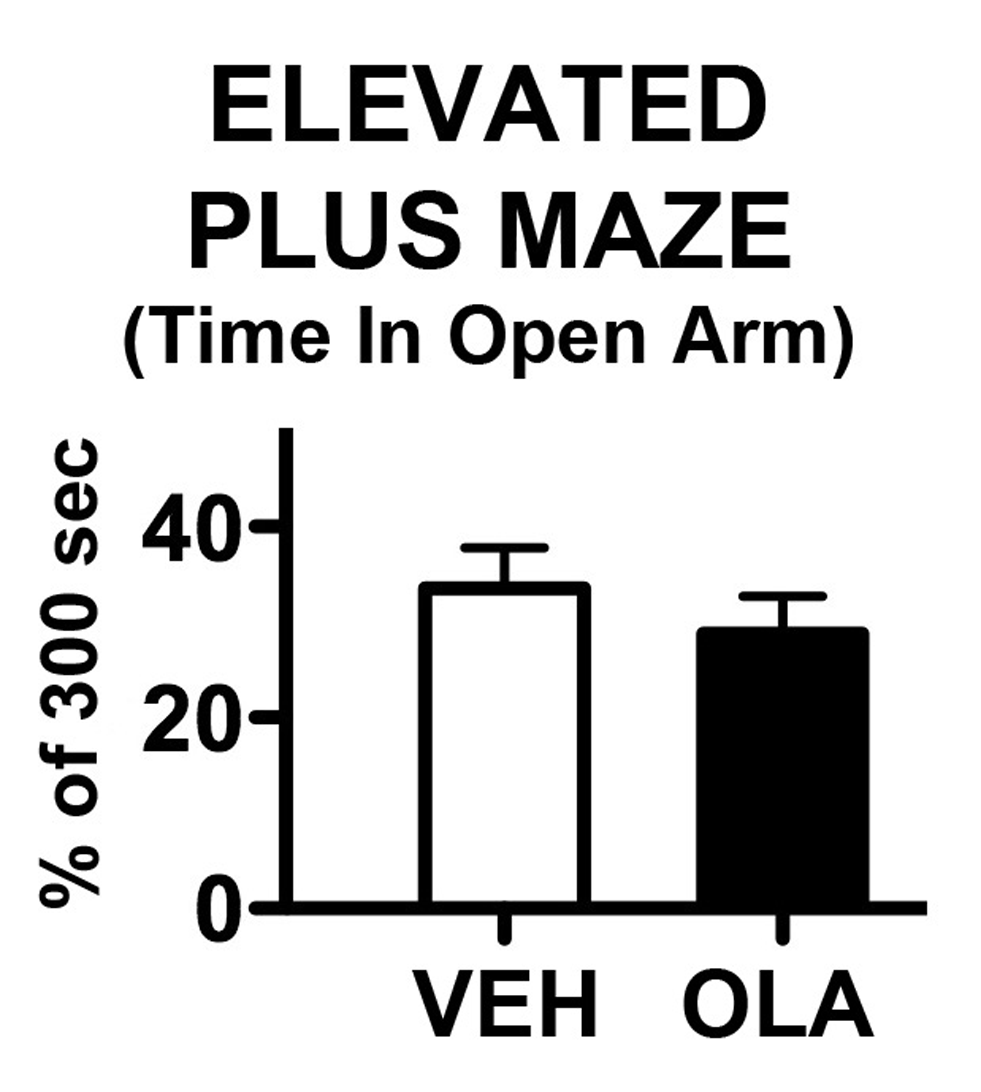

Supplement: Figure S3 — Elevated plus maze. The EPM, a “+” shaped maze with 2 closed- and 2 open arms, was used to test anxiety-like behavior. Each arm measured 113×10 cm. The maze was 88 cm above the floor. Rats were placed in the center of the maze facing a closed arm and were allowed to explore freely for 5 min. Time spent in the open arms is an inverse measure of an anxiety-like phenotype in rodents. There was no significant effect of treatment (T[1,18] = 0.664; p = 0.426). Error bars are standard error of the mean. (TIF) [file pone.0057308.s003.tif]

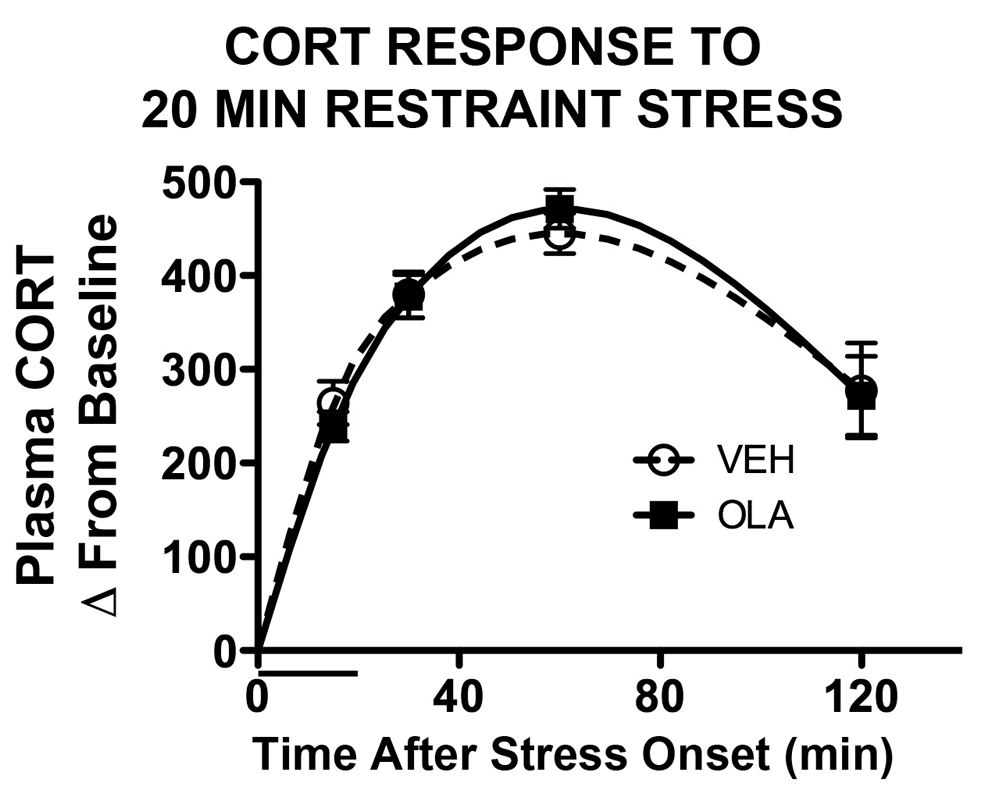

Supplement: Figure S4 — Hypothalamo-pituitary-adrenal (HPA) axis responses to mild, transient stress. All test procedures were initiated starting ≥1.5 h after lights on and were completed no later than 5.5 h after lights on, to guarantee that all measurements were made during the phase of the diurnal cycle when ACTH and corticosterone (CORT) levels are constant and at their nadir. For a week prior to testing, subjects were brought daily to the procedure room, to habituate them to being moved from the adjacent colony room. On the first day of testing, baseline blood samples were obtained by tail nick immediately after subjects were brought into the procedure room. After a 72 h recovery period, subjects were placed in a plastic restrainer immediately after being brought into the procedure room; restraint was maintained for the first 20 min of the 2 h test; for the remainder, subjects were placed in a holding cage, after which they were returned to their home cages. Blood samples were obtained by tail nick at 15, 30, 60 and 120 minutes following the onset of restraint. Samples (∼300 µl) were typically obtained ≤1 min (always ≤2 min) post-nick. Blood was collected in EDTA-coated tubes and maintained at 4°C during testing. At the completion of testing, blood samples were centrifuged at 2000×g for 10 min at 4°C and the plasma was aliquoted into Eppendorf tubes and stored at −80° C. Measurements of plasma CORT were obtained in duplicate using I125 radioimmunoassay performed at the University of Virginia Center for Research in Reproduction Ligand Assay and Analysis Core Laboratory (http://www.medicine.virginia.edu/research/institutes-and-program/crr/lab-facilities). The effects of treatment, time and their interaction were determined by repeated measures ANOVA.There was no significant treatment effect on baseline plasma CORT concentration (not illustrated; t[22] = 1.038; p>0.05; OLA = 114.1±17.3 ng/mL; VEH = 142.9±22.2 ng/mL). Plasma CORT concentration (baseline subtracted) showed a significant ov [file pone.0057308.s004.tif]

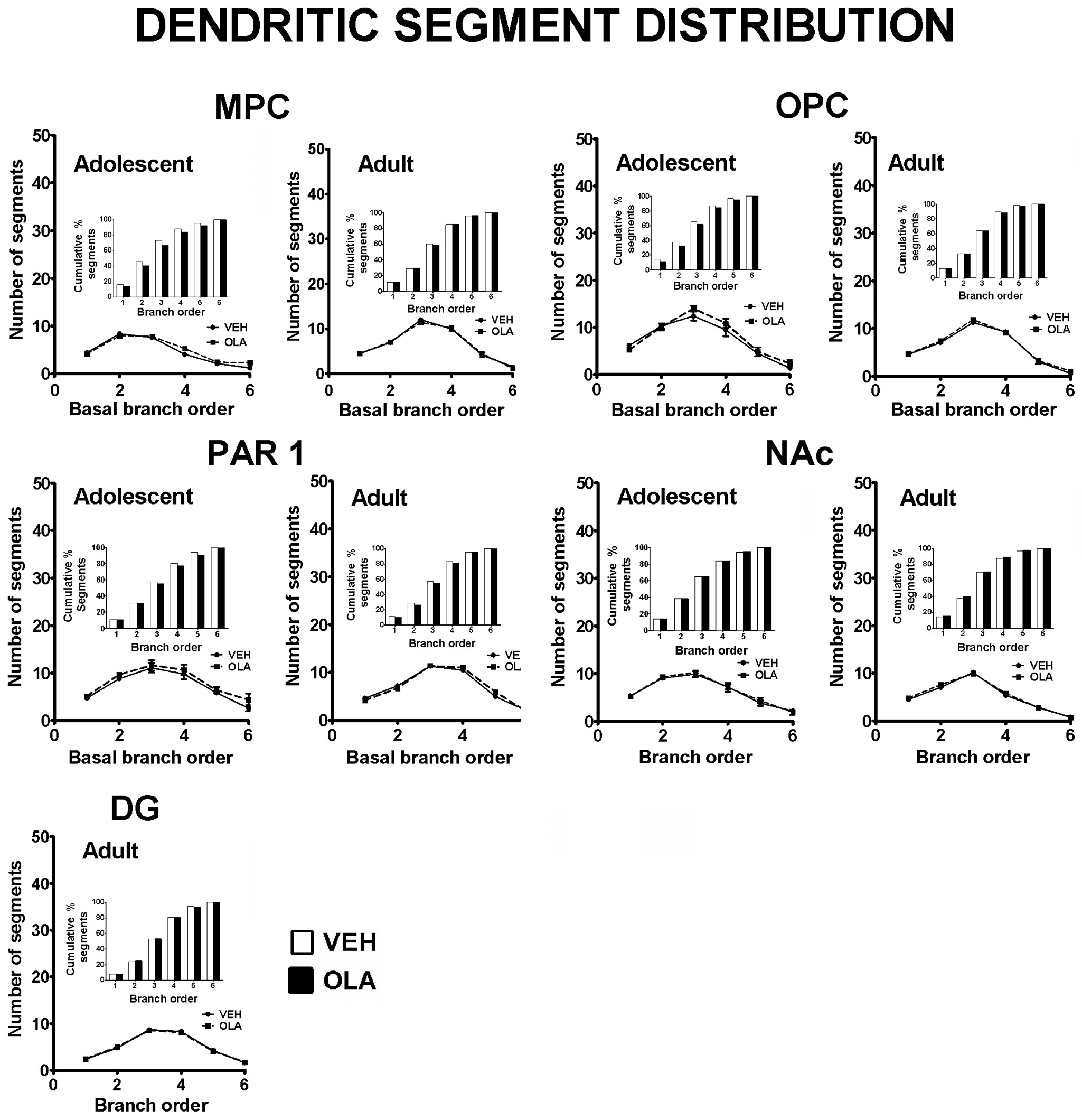

Supplement: Figure S5 — Distribution of dendritic segments among branches of different order. We made a supplemental analysis of our data on the number of dendritic segments of each order because changes in the distribution of branching among segments of different order, which would not be detected by analysis of the total number of branches, can affect the integration of inputs converging on the dendritic tree. The curves at the bottom of each frame show the number of segments of each branch order for the ROI and age indicated. The cumulative distributions in the insets show the percentage of branches at or below each branch order. Error bars are standard error of the mean. Two-way repeated measures ANOVA of the number of segments of each order did not reveal any significant effects of treatment or branch order X treatment interaction. (TIF) [file pone.0057308.s005.tif]
